# Assessment of the causal relevance of ECG parameters for risk of atrial fibrillation: A mendelian randomisation study

**DOI:** 10.1371/journal.pmed.1003572

**Published:** 2021-05-13

**Authors:** Parag Ravindra Gajendragadkar, Adam Von Ende, Maysson Ibrahim, Elsa Valdes-Marquez, Christian Fielder Camm, Federico Murgia, Alexander Stiby, Barbara Casadei, Jemma C. Hopewell

**Affiliations:** 1 CTSU, Nuffield Department of Population Health, BHF Centre of Research Excellence, University of Oxford, Oxford, United Kingdom; 2 Division of Cardiovascular Medicine, BHF Centre of Research Excellence, University of Oxford, Oxford, United Kingdom; Chinese University of Hong Kong, CHINA

## Abstract

**Background:**

Atrial electrical and structural remodelling in older individuals with cardiovascular risk factors has been associated with changes in surface electrocardiographic (ECG) parameters (e.g., prolongation of the PR interval) and higher risks of atrial fibrillation (AF). However, it has been difficult to establish whether altered ECG parameters are the cause or a consequence of the myocardial substrate leading to AF. This study aimed to examine the potential causal relevance of ECG parameters on risk of AF using mendelian randomisation (MR).

**Methods and findings:**

Weighted genetic scores explaining lifelong differences in P-wave duration, PR interval, and QT interval were constructed, and associations between these ECG scores and risk of AF were estimated among 278,792 UK Biobank participants (mean age: 57 years at recruitment; 19,132 AF cases). The independent genetic variants contributing to each of the separate ECG scores, and their corresponding weights, were based on published genome-wide association studies. In UK Biobank, genetic scores representing a 5 ms longer P-wave duration or PR interval were significantly associated with lower risks of AF (odds ratio [OR] 0.91; 95% confidence interval [CI]: 0.87–0.96, *P* = 2 × 10^−4^ and OR 0.94; 95% CI: 0.93–0.96, *P* = 2 × 10^−19^, respectively), while longer QT interval was not significantly associated with AF. These effects were independently replicated among a further 17,931 AF cases from the AFGen Consortium. Investigation of potential mechanistic pathways showed that differences in ECG parameters associated with specific ion channel genes had effects on risk of AF consistent with the overall scores, while the overall scores were not associated with changes in left atrial size. Limitations of the study included the inherent assumptions of MR, restriction to individuals of European ancestry, and possible restriction of results to the normal ECG ranges represented in UK Biobank.

**Conclusions:**

In UK Biobank, we observed evidence suggesting a causal relationship between lifelong differences in ECG parameters (particularly PR interval) that reflect longer atrial conduction times and a lower risk of AF. These findings, which appear to be independent of atrial size and concomitant cardiovascular comorbidity, support the relevance of varying mechanisms underpinning AF and indicate that more individualised treatment strategies warrant consideration.

## Abstract 2

In a Mendelian randomization analysis, Parag Gajendragadkar and colleagues investigate associations between genetically-predicted EEG parameters and risk of atrial fibrillation among UK Biobank participants.

## Author summary

**Why was this study done?:**

- Atrial fibrillation (AF) is the most common arrhythmia worldwide and associated with higher risk of stroke, dementia, heart failure, and death.
- Incomplete understanding of the underlying substrates for AF has hampered therapeutic advances and effective risk stratification, with current anti-arrhythmic drug therapy and a variety of catheter ablation strategies for AF still showing high medium-term failure rates and no material effects on the risk of stroke.
- Observational studies have suggested associations between electrocardiographic (ECG) parameters and risk of AF, but these associations may be subject to confounding and reverse causality, and thus the causal relevance of ECG parameters for AF remains unclear.

**What did the researchers do and find?:**

- We used a mendelian randomisation (MR) approach to examine the potential causal relevance of lifelong differences in ECG parameters for AF among approximately 300,000 individuals in UK Biobank.
- Unexpectedly, we found evidence supporting a causal association between lifelong differences in ECG parameters representing longer atrial conduction times within the normal range and a lower risk of AF.
- The relationship between ECG parameters and AF appeared to be independent of atrial size and cardiovascular comorbidities.

**What do these findings mean?:**

- These findings further emphasise that substantial variation in the mechanisms underpinning AF exists and warrants the consideration of more individualised treatment strategies.

## Introduction

Atrial fibrillation (AF) is a heterogeneous disease with multiple proposed pathophysiological pathways [1]. Incomplete understanding of the underlying substrates for AF has hampered therapeutic advances and effective risk stratification, with current anti-arrhythmic drug therapy and a variety of catheter ablation strategies for AF still showing high medium-term failure rates and no significant effects on the risk of stroke [2,3].

Some of the proposed mechanisms for the development and maintenance of AF involve alterations in atrial electrical and structural characteristics predisposing to arrhythmogenesis [4]. Simple electrocardiographic (ECG) parameters such as P-wave duration [5–7], PR interval [8–10], and QT interval [11–14] have been associated with incident AF in large observational studies, where higher risks of AF were observed at both short and long values (i.e., a “U-shaped relationship”). If causal, these findings may refine our mechanistic understanding of atrial arrhythmias and inform therapeutic strategies. However, observational studies are potentially subject to confounding, commonly by age and comorbid cardiac conditions affecting both ECG parameters and the risk of developing AF. In particular, P-wave and PR interval duration have been shown to be associated with hypertension [15], ischaemic heart disease, and heart failure [16], all of which are established risk factors for AF. Furthermore, the association between ECG parameters and AF may be subject to reverse causality, since having a history of AF or being treated with rate or rhythm control drugs may alter ECG parameters. These potential biases limit the value of observational studies for making causal inferences.

Mendelian randomisation (MR) can circumvent these limitations by using genetic variants as proxies for a trait and then estimating their association with an outcome of interest. As the genetic variants are randomly allocated at conception, this produces a “naturally randomised” experiment by which groups with differences in lifelong levels of a trait would be anticipated to be balanced on potential confounders.

The objective of the study was to assess the potential causal relevance of ECG parameters for the risk of AF using MR techniques, with the aim of improving our understanding of the mechanisms underlying AF. We used genetic variants associated with lifelong differences in cardiac electrical parameters and examined their associations with AF among approximately 300,000 individuals in UK Biobank. Additionally, we investigated relationships between ECG parameters and other atrial arrhythmias, specifically non-AF supraventricular tachycardias (SVTs), which are generally considered to be mechanistically different from AF [17].

## Methods

This study is reported in accordance with the Strengthening the Reporting of Observational Studies in Epidemiology (STROBE) guidelines (**S1 Checklist**) [18] and adheres to the key principles of STROBE-MR [19]. No formal protocol was prespecified, but the aims of the study were formalised as well as the methods for generation of the main ECG genetic scores, AF case definitions, and main sensitivity analyses, prior to the analyses being conducted. Post hoc analyses examining ion channel scores, left atrial volumes, and SVT were later performed to ascertain further biological insights and robustness of the study results. Further sensitivity analyses were undertaken in response to peer review (as detailed subsequently).

### Study populations and outcome definitions

#### UK Biobank

Primary analyses were conducted using individual-level data in UK Biobank, a population-based study including approximately 500,000 individuals from the United Kingdom, who were 40 to 69 years old at the time of recruitment (2006 to 2010). UK Biobank includes extensive baseline data including medical history as well as prospective linkage to electronic healthcare records. Further information regarding the UK Biobank data as well as detailed genotyping procedures have been reported elsewhere [20,21]. In the present study, a total of 278,792 participants were included in the primary analyses, after exclusion of samples failing bioinformatic quality control (extreme heterozygosity, missing rate on autosomes of >0.02, and sex mismatch between reported and genetically inferred), those of non-white British ancestry, and related participants. Resting 12-lead ECGs and interval measurements (using Cardiosoft v6 program, GE Healthcare, Chicago, USA) were available in 15,365 of these participants and in 13,314 of those after excluding anyone who had AF on ECG or AF identified from self-reports at baseline or electronic healthcare records. Cardiac magnetic resonance imaging (MRI) data on left atrial volumes indexed to body surface area were available in 2,581 participants after excluding 415 participants with either poor quality images or AF identified from self-reports or electronic healthcare records. Details of the cardiac MRI protocol have been reported elsewhere [22]. A flow diagram detailing how the different datasets were generated is shown in **Fig A** in **S1 Appendix**.

#### Defining AF, SVT, and other outcomes in UK Biobank

We identified 19,132 cases of AF or flutter from electronic healthcare records (97% of cases) or self-reported at nurse-led interviews at baseline (3% of cases). Cases defined using healthcare records had at least 1 diagnostic code from national in-patient hospital data statistics (International Statistical Classification of Diseases-10th Revision [ICD-10] codes), procedure codes (national classification of interventions and procedure [OPCS-4]), or national death records. A total of 4,805 participants were identified as having “lone” AF, defined as AF without known coronary heart disease, heart failure, hypertension, or diabetes (based on self-reported medical conditions or electronic healthcare records available at baseline, as well as electronic healthcare record diagnoses up to 1 year after the first recorded AF diagnosis). Furthermore, 2,884 cases of non-AF SVT were identified using diagnostic codes that excluded AF or flutter and that had been previously validated in an external cohort [23]. Details of definitions of codes used for outcomes are listed in **Tables A** and **B** in **S1 Appendix**. Please see **Supporting Methods** in **S1 Appendix** for further details about sources of data.

### Selection of genetic variants

For each individual ECG parameter in turn, genetic variants with genome-wide significant associations (*P* < 5 × 10^−8^) were selected from published studies [24–27], none of which included UK Biobank participants, and independent genetic variants were identified using a clumping approach (linkage disequilibrium r^2^ < 0.01 within a ± 250-kb window). Unless otherwise stated, the coded allele was defined as the allele associated with a longer ECG interval. The P-wave duration score comprised 8 variants, the PR interval score comprised 52 variants, and the QT interval score comprised 54 variants (**Tables C–E** in **S1 Appendix**). An overview of the selection process is presented in **Fig B** in **S1 Appendix**.

To examine the specificity of any findings to electrical pathways, for each ECG parameter, we identified a subset of variants that were annotated to gene loci for known ion channels (e.g., *SCN5A*, *SCN10A*, *KCNQ1*, and *KCNJ2*). Full details of the selection process and identified variants are available in the **Supporting Methods** and in **Tables C-E** in **S1 Appendix**.

### Statistical analysis

#### UK Biobank

In UK Biobank, separate genetic scores for P-wave duration, PR interval, and QT interval were calculated for each participant by summing the number of coding alleles of each variant, weighted by their published per-allele effect size on the ECG parameter (in milliseconds; ms). The distributions of the different ECG genetic scores are shown in **Fig C** in **S1 Appendix**. Associations between the genetic scores and 12-lead ECG parameters were estimated using linear regression and associations between genetic scores and risk of AF and SVT using logistic regression. All analyses involving the genetic scores were adjusted for sex, genotyping array, and 40 principal components of ancestry (as provided by UK Biobank) [20]. Effects of the ECG genetic scores on risk of AF and SVT are reported per 5 ms difference in ECG score. Confidence intervals (CIs) based on floated variances are presented in figures for variables with more than 2 levels in order to allow direct comparisons between different groups (avoiding restriction to a single arbitrary reference group). Estimates and standard CIs are given for direct 2-way comparisons. To allow for multiple testing, 2-tailed *P* values <0.01 were considered significant, and *P* values <0.05 were considered suggestive.

#### Power calculations

The variance explained by each genetic score for each ECG parameter was estimated (P-wave duration: 1.8%; PR interval: 5.0%; QT interval: 5.0%) and used to inform power calculations with details described in the **Supporting Methods** in **S1 Appendix**. *F* statistics were computed to quantify the strength of the selected genetic variants, and all were well above the F>10 threshold typically recommended to avoid weak instrument bias in MR analyses [28]. The different ECG scores had between 80% and 99% power to detect a 15% effect on risk of AF in UK Biobank per standard deviation unit of the ECG parameters (**Table F** in **S1 Appendix**).

#### External replication dataset

Replication analyses were performed using publicly available summary data from the AFGen Consortium genome-wide meta-analysis [29] accessed via the Cardiovascular Disease Knowledge Portal (2017 AFGen genome-wide association study [GWAS] [30]). Details of the contributing studies, phenotyping, and genotyping processes have been published previously and did not include any UK Biobank participants [29]. Estimates of the log odds of AF (per-allele) for individual genetic variants among a total of 17,931 AF cases and 115,142 controls of primarily (approximately 90%) European ancestry were available. Based on these data, we estimated causal effects for each ECG score on risk of AF using the fixed-effects inverse variance weighted method for summarised data. Meta-analyses of the AFGen and UK Biobank estimates were performed using fixed-effects methods.

#### Sensitivity analyses in UK Biobank

Causal inferences from MR studies are subject to instrumental variable assumptions [31]. In particular, MR can be sensitive to pleiotropy, which can occur when genetic variants potentially affect independent biological pathways for risk factors associated with the outcome. In the primary analysis method, all genetic variants are assumed to be valid instruments. Therefore, we conducted sensitivity analyses to establish the robustness of the MR results including the fixed-effects inverse variance weighted method combining individual causal estimates across the selected genetic variants (with random effects inverse variance weighted results added in response to peer review); the weighted median approach (in which 50% of the genetic variants are permitted to be invalid instrumental variables); the weighted mode approach (which allows the majority of variants to be invalid provided the most common causal effect estimate is a consistent estimate of the true causal effect); and MR–Egger (in which all genetic variants are permitted to be invalid instrumental variables, provided that the pleiotropic and risk factor effects of the variants are independently distributed) [31,32]. Further analyses of each genetic ECG score were also undertaken after exclusion of outlier variants identified using the MR-PRESSO outlier detection method [33]. In addition, to further explore potential pleiotropic associations, the PhenoScanner [34] search tool was used (see **Supporting Methods** in **S1 Appendix**) to identify published associations with each of the genetic variants (and those in close linkage disequilibrium) selected for the ECG parameter scores.

Finally, in order to investigate whether being at higher risk of AF may have causal effects on ECG parameters, bidirectional MR analyses were conducted using a genetic score for risk of AF (based on variants with published genome-wide significant associations with AF, using weights excluding UK Biobank participants, and defined using comparable methodology as described above [35]) to ascertain the direction of effect. Further methodological details are described in the **Supporting Methods** in **S1 Appendix**, and details of genetic variants selected are provided in **Table G** in **S1 Appendix**.

All analyses were performed using SAS (v 9.4, SAS Institute, Cary, USA) and R (v3.5.2, The R Foundation for Statistical Computing, Vienna, Austria, https://www.r-project.org/). Additional software details are provided in the **Supporting Methods** in **S1 Appendix**.

### Standard protocol approvals, registrations, and patient consents

This research has been conducted using the UK Biobank Resource under Application Number 14568. All procedures and data collection in UK Biobank were approved by the UK Biobank Research Ethics Committee (reference number 11/NW/0274) with participants providing full written informed consent for participation in UK Biobank and subsequent use of their data for approved applications. Publicly available non-identifiable summary data from the AFGen Consortium was used in this study. The multiple contributory studies to the AFGen Consortium (https://www.afgen.org/) have their own participant consent procedures.

## Results

### UK Biobank participant characteristics

In UK Biobank, participants with AF were older, more likely to be male, and had a higher prevalence of cardiovascular risk factors than those without AF (**Table 1**). Of the 259,660 control subjects without a history of AF, 13,314 participants had a 12-lead ECG. Within this cohort, the mean and standard deviation of P-wave duration was 97 ± 16 ms (95% of measured P-wave durations between 65 and 129 ms); for PR interval, 163 ± 27 ms (95% of measured PR intervals between 109 and 217 ms); and for Bazett corrected QT_c_, 420 ± 26 ms (95% of measured QT_c_ between 368 and 472 ms).

**Table 1:** Baseline characteristics of participants with AF in UK Biobank.

Characteristic	No AF	AF
Number of participants	259,660	19,132
Age at recruitment (year)	56.6 ± 8.0	62.2 ± 5.9
Male sex	118,596 (45.7)	12,662 (66.2)
Body mass index (kg/m^2^)	27.3 ± 4.7	29.0 ± 5.3
Coronary heart disease	19,297 (7.4)	7,332 (38.3)
Heart failure	2,840 (1.1)	3,826 (20.0)
Hypertension	80,806 (31.1)	15,862 (66.5)
Diabetes	16,056 (6.2)	3,270 (17.1)
“Lone” AF	0 (0)	4,805 (25.1)
Other SVTs	1,164 (0.4)	1,720 (9.0)

Population restricted to unrelated white British ancestry (see **Methods** for full details). Continuous variables are presented as mean ± standard deviation and categorical variables as number (%). “Lone” AF is defined as AF without known coronary heart disease, heart failure, hypertension, or diabetes (see **Methods** for full definition).

AF, atrial fibrillation; SVT, supraventricular tachycardia.

### Association of genetic scores with measured 12-lead ECG parameters

**Fig 1:**
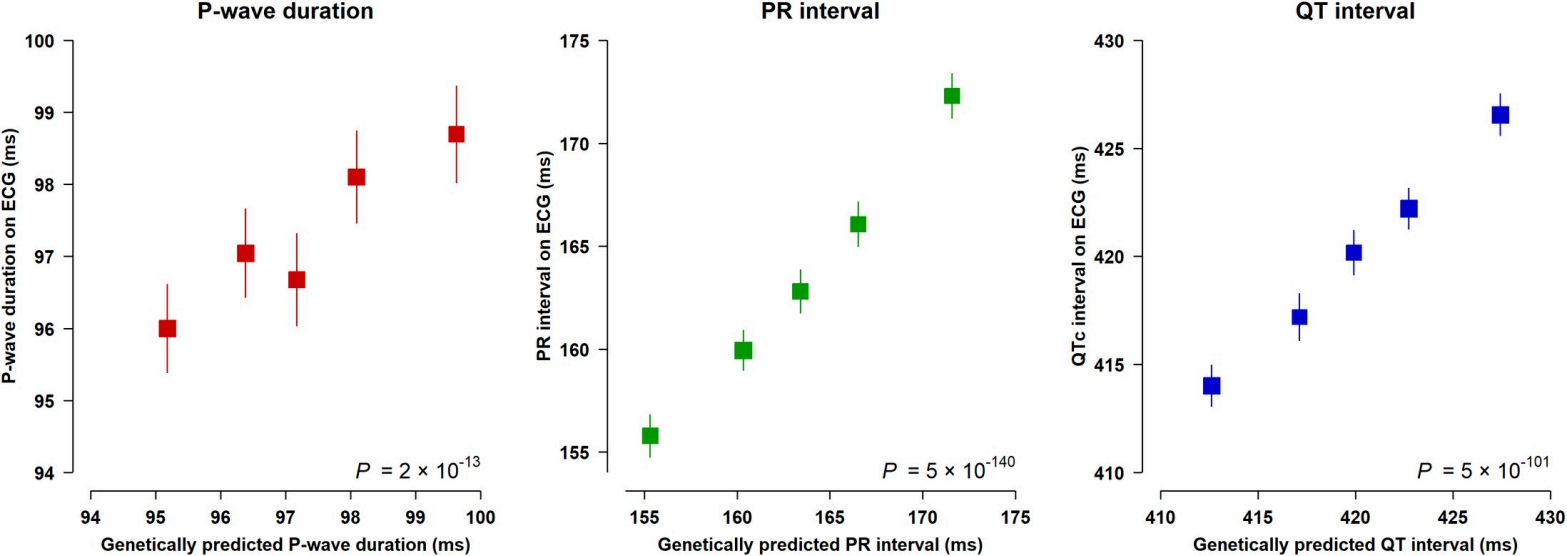
Associations between genetic scores and 12-lead ECG parameters in UK Biobank. Association between quintiles of genetic scores for ECG parameters (defined in non-AF cases) and 12-lead ECG parameters in 13,314 participants in UK Biobank. Boxes represent mean values with their size inversely proportional to variance, and lines represent 95% CIs. A constant representing the population mean nongenetically predicted ECG interval was added to all genetic score values (the population mean of measured 12-lead ECG parameter excluding the genetic contribution; 93.2 ms for P-wave duration, 93.5 ms for PR interval, and 360.7 ms for QT interval) for display purposes. *P* calculated across the genetic score using linear regression adjusting for sex, genotyping array, and 40 principal components of ancestry. AF, atrial fibrillation; CI, confidence interval; ECG, electrocardiographic.

To illustrate the association between each genetic score and corresponding ECG parameter, a required assumption of MR analyses, we used the subset of 13,314 UK Biobank participants with a 12-lead ECG and no history of AF (to reduce potential for reverse causality). Each genetic score was significantly associated with the corresponding measured 12-lead ECG parameter (5 × 10^−140^ < *P* < 2 × 10^−13^), predicting these within normal reference ranges (**Fig 1**).

### Genetically predicted differences in ECG parameters and risk of AF

**Fig 2:**
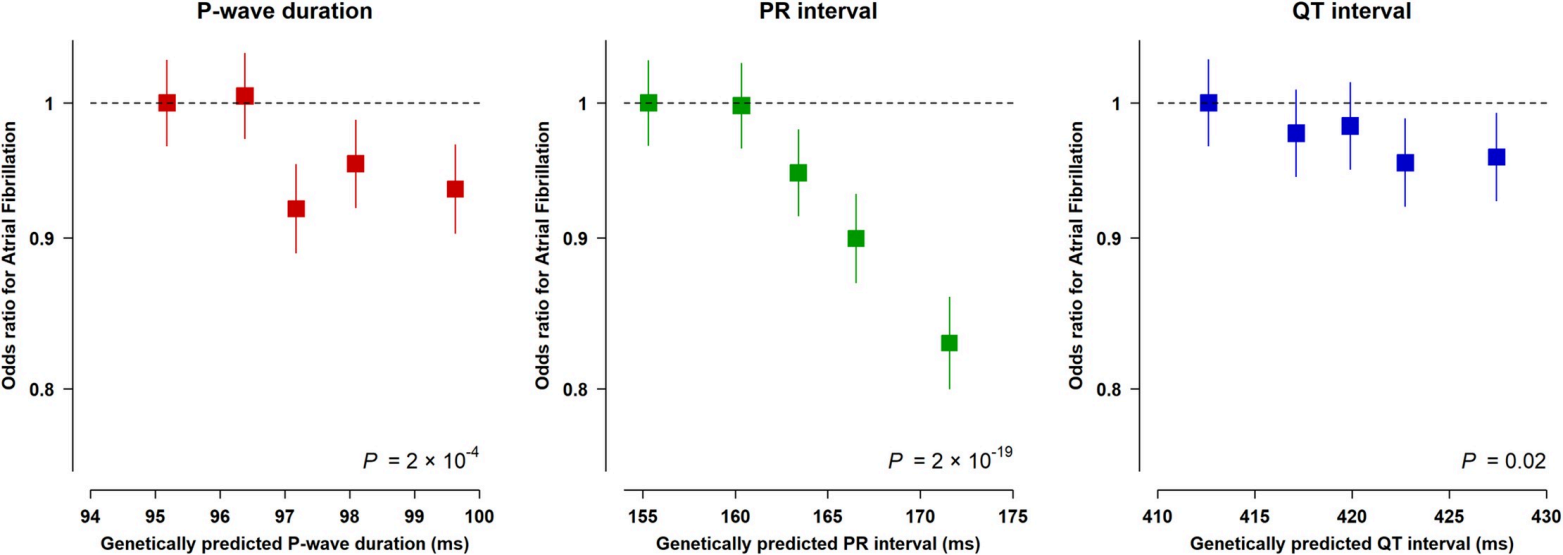
Effects of genetically predicted ECG parameters on risk of AF in UK Biobank. ORs for AF per quintile of genetic score (defined in non-AF cases) in 278,792 participants in UK Biobank. Boxes represent effect estimates with their size inversely proportional to variance. Solid lines represent 95% CIs calculated using floating absolute risks. ORs are adjusted for genotyping array, sex, and 40 principal components of ancestry. *P* calculated across continuous genetic score values adjusting for sex, genotyping array, and 40 principal components of ancestry. AF, atrial fibrillation; CI, confidence interval; OR, odds ratio.

In UK Biobank, a genetically predicted longer P-wave duration was associated with a lower risk of AF, with a 6% lower risk of AF in the top versus bottom quintile of the P-wave genetic score (odds ratio [OR] 0.94; 95% CI: 0.89 to 0.98, *P* = 5 × 10^−3^; **Fig 2**) and 9% lower risk of AF per 5 ms longer P-wave duration (OR 0.91; 95% CI: 0.87 to 0.96, *P* = 2 × 10^−4^). Genetically predicted differences in PR interval were strongly associated with AF, with a 17% lower risk of AF in the top versus bottom quintile of the PR interval genetic score (OR 0.83; 95% CI: 0.79 to 0.87, *P* = 9 × 10^−15^; **Fig 2**) resulting in a 6% lower risk of AF per 5 ms longer PR interval (OR 0.94; 95% CI: 0.93 to 0.96, *P* = 2 × 10^−19^). To explore the independent effect of PR interval after consideration of genetically predicted P-wave duration, we repeated the regression analyses after adjusting for the P-wave duration score; the effect of PR interval was unchanged and remained significantly associated with AF (OR 0.94 per 5 ms longer PR interval, 95% CI: 0.93 to 0.96, *P* = 3 × 10^−16^). Differences in QT interval were not associated with statistically significant differences in risk of AF (OR 0.96 for top versus bottom quintile; 95% CI: 0.91 to 1.00, *P =* 0.08; **Fig 2**), albeit a statistically suggestive 2% lower risk of AF per 5 ms longer QT interval was observed (OR 0.98; 95% CI: 0.97 to 1.00, *P* = 0.02). The effects seen for all ECG parameters were similar in both males and females.

**Fig 3:**
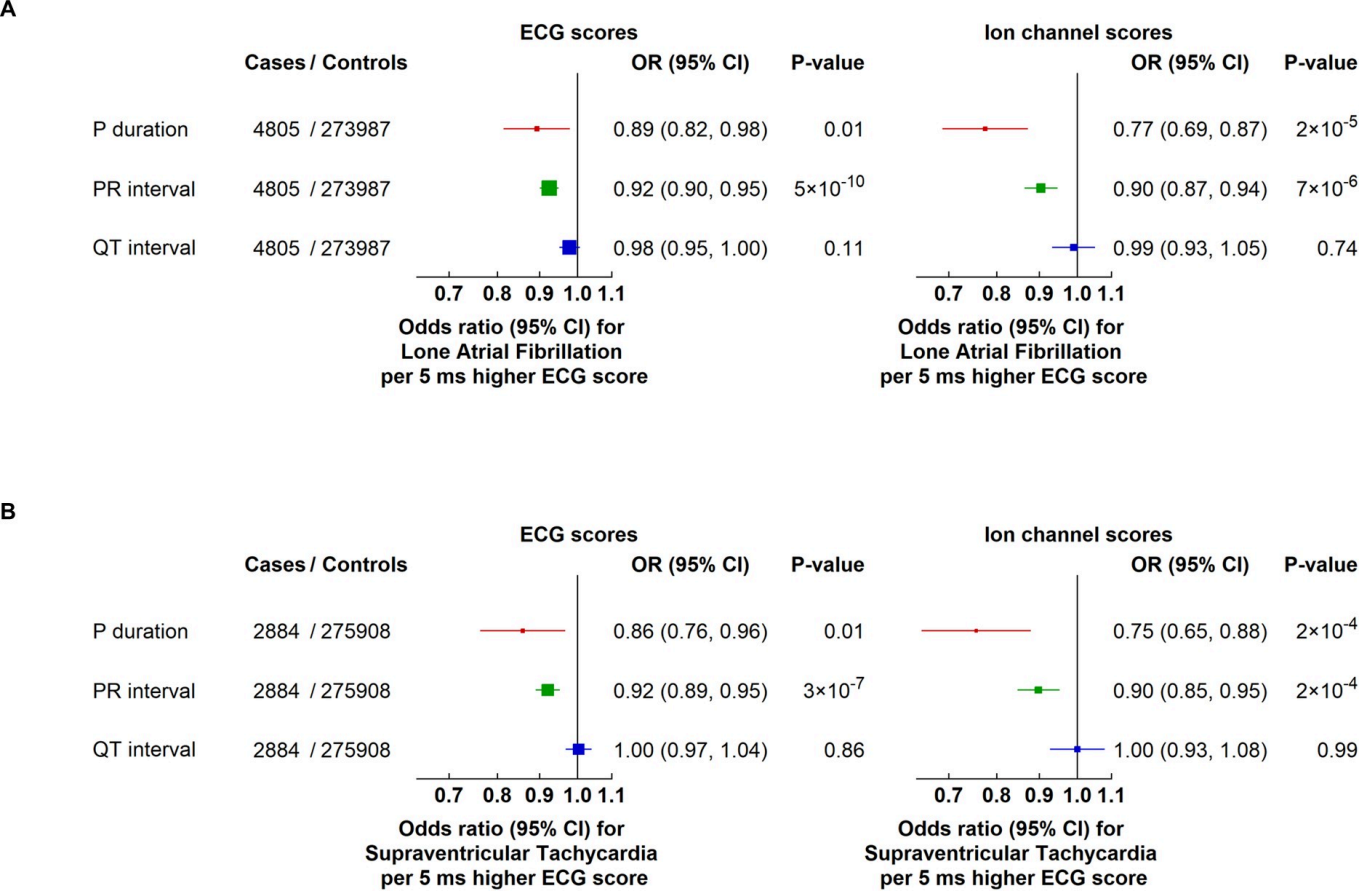
Effects of genetically predicted ECG parameters on the risk of lone AF and SVT in UK Biobank. Effects of genetically predicted ECG parameters on risk of (A). “Lone” AF and (B). SVTs in UK Biobank. Ion channel scores for each ECG parameter include only genetic variants annotated to known ion channel genes. Boxes represent point estimates of effect per 5 ms higher ECG score with sizes inversely proportional to their variance, and lines represent 95% CIs. ORs are adjusted for genotyping array, sex, and 40 principal components of ancestry. AF, atrial fibrillation; CI, confidence interval; ECG, electrocardiographic; OR, odds ratio; SVT, supraventricular tachycardia.

To explore whether the association between ECG scores and AF was affected by underlying cardiovascular comorbidities, we also examined associations with “lone” AF (as defined in the **Methods**), for which comparable effects were observed. In particular, as well as being associated with all AF, genetically predicted differences in P-wave duration and PR interval were also associated with lower risks of lone AF (OR per 5 ms longer P-wave duration: 0.89; 95% CI: 0.82 to 0.98, *P* = 0.01 and OR per 5 ms longer PR interval: 0.92; 95% CI: 0.90 to 0.95, *P* = 5 × 10^−10^). There was no significant association with QT interval (OR 0.98; 95% CI: 0.95 to 1.00, *P* = 0.11; **Fig 3A**, **Fig D** in **S1 Appendix**).

### Genetically predicted differences in ECG parameters and risk of SVT

To explore if our findings also applied to SVT phenotypes, which are generally regarded as being mechanistically different to AF [17], we investigated the relationship between the ECG genetic scores and risk of (non-AF) SVT among 2,884 SVT and 275,908 non-SVT cases. Compared to those with AF, participants with other SVTs were younger, less likely to be male, and had fewer cardiovascular comorbidities (**Table H** in **S1 Appendix**).

As for AF, genetic scores associated with 5 ms longer P-wave duration and PR interval were also associated with a lower risk of SVT (OR 0.86; 95% CI: 0.76 to 0.96, *P* = 0.01 and OR 0.92; 95% CI: 0.89 to 0.95, *P* = 3 × 10^−7^, respectively). Furthermore, the association of PR interval with risk of SVT was unchanged after adjusting for the P-wave duration score (OR 0.92; 95% CI: 0.89 to 0.96, *P* = 9 × 10^−6^ per 5 ms longer score). In contrast, and consistent with the lack of relationship between QT interval and AF, there was no significant association of QT interval with SVT (OR 1.00; 95% CI: 0.97 to 1.04, *P* = 0.86; **Fig 3B**). Comparable associations were observed after exclusion of participants with any AF diagnosis.

### Genetically predicted ECG parameter score associations with left atrial size on cardiac MRI

ECG parameters may be affected by atrial chamber size, which could also alter the risk of AF. To investigate whether differences in atrial chamber size may have contributed to our findings, we tested the associations of our ECG genetic scores with indexed biplanar left atrial volumes in 2,581 participants with cardiac MRI data and no history of AF. The scores were not associated with differences in left atrial volumes (−0.37 ml/m^2^ per 5 ms longer P-wave duration; 95% CI: −0.84 to 1.60, *P* = 0.54; 0.04 ml/m^2^ per 5 ms longer PR interval; 95% CI: −0.28 to 0.38, *P* = 0.79; and 0.18 ml/m^2^ per 5 ms longer QT interval; 95% CI: −0.18 to 0.53, *P* = 0.34), suggesting that the effects of the genetically predicted differences in atrial ECG parameters on risk of AF and SVT were not associated with left atrial size.

### Genetically predicted ECG parameters, via ion channel pathways, and risk of AF, lone AF, and SVT

**Fig 4:**
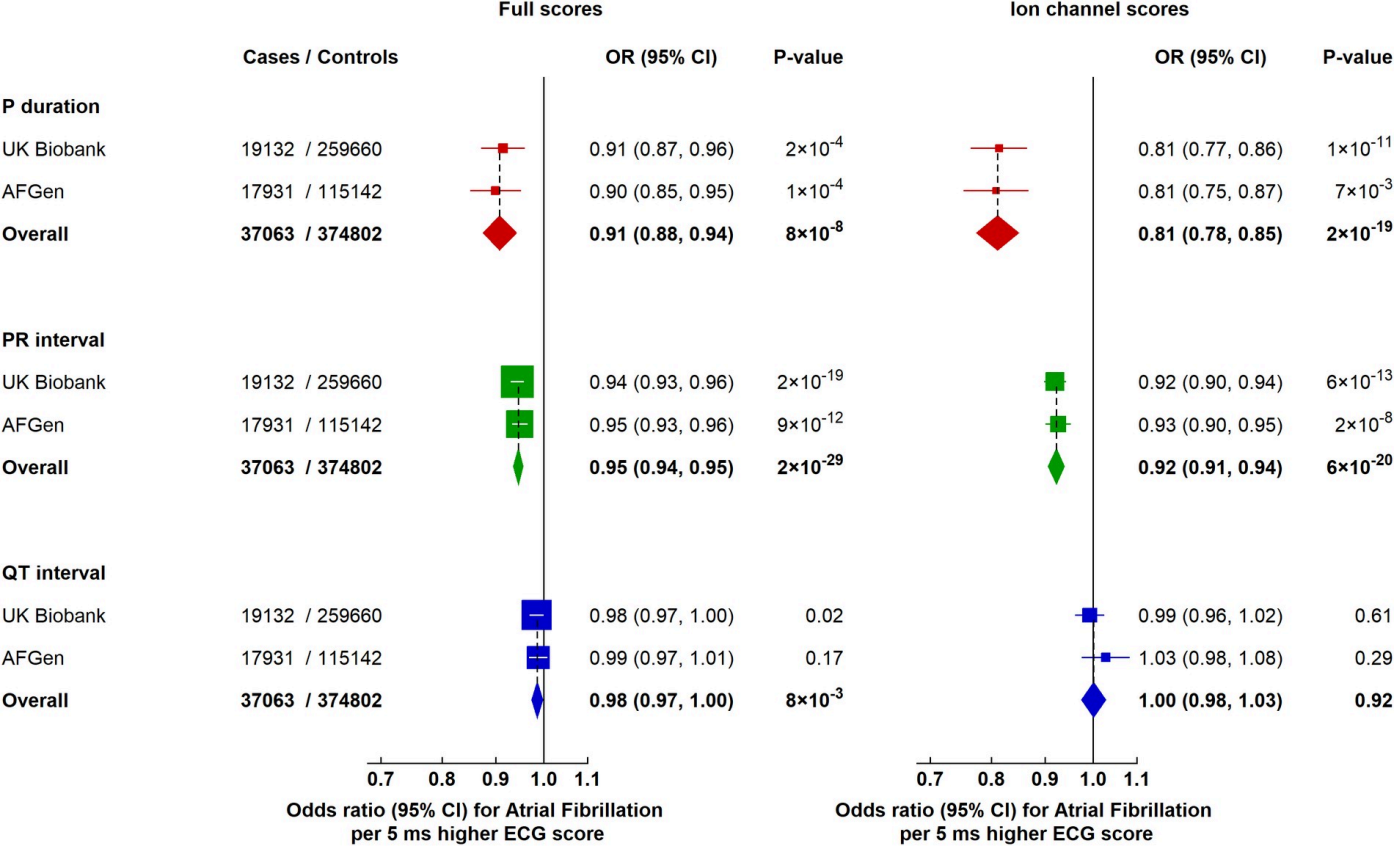
Effects of genetically predicted ECG parameters on risk of AF in UK Biobank and AFGen. Ion channel scores for each ECG parameter include only genetic variants annotated to known ion channel genes. Boxes represent point estimates of effect per 5 ms higher ECG score, diamonds represent meta-analysis estimates, with sizes inversely proportional to their variance, and lines represent 95% CIs. UK Biobank derived ORs are adjusted for sex, genotyping array, and 40 principal components of ancestry. ORs for AFGen were calculated using inverse variance weighted fixed-effects methods from published summary data. AF, atrial fibrillation; CI, confidence interval; ECG, electrocardiographic; OR, odds ratio.

To investigate the relevance of specific electrical mechanisms for AF or SVT, we examined the effects of genetic scores for the different ECG parameters restricted to variants annotated to known ion channel genes (defined using independent databases—see **Methods**). Variants associated with longer P-wave duration and PR interval were similarly associated with lower risks of AF, lone AF, and SVT, with no association with atrial arrhythmia seen for variants in ion channel genes associated with QT interval, and directionally consistent with estimates from the genetic scores that included all variants (**Figs 3** and **4**). Overall, these results suggest that changes in ion channel pathways (along with other pathways) contribute to the associations of the atrial ECG parameters on risk of AF and SVT.

### Independent replication and meta-analysis

The results for the associations of genetic ECG parameter scores with risk of AF were independently replicated in data from the AFGen Consortium [29], with estimates similar to those seen in UK Biobank for the overall scores as well as the ion channel scores (**Fig 4**). Overall, a meta-analysis of a total of 37,063 cases of AF and 374,802 controls showed that a 5 ms longer genetically predicted P-wave duration, PR interval, and QT interval were each associated with a lower risk of AF (OR 0.91; 95% CI: 0.88 to 0.94, *P* = 8 × 10^−8^; OR 0.95; 95% CI: 0.94 to 0.95, *P* = 2 × 10^−29^, and OR 0.98; 95% CI: 0.97 to 1.00, *P* = 8 × 10^−3^, respectively; **Fig 4**).

### Sensitivity analyses

#### Genetically predicted risk of AF and effect on 12-lead ECG parameters

To assess if a higher genetic risk of AF was associated with differences in measured ECG parameters, (potential bidirectional effect), the effects of a genetic AF risk score (generated as described in the **Methods**) on 12-lead ECG parameters were examined in 13,314 individuals in UK Biobank with ECG data and no history of AF. Overall, there were no significant associations between genetically predicted AF risk and PR interval or QTc interval, but higher genetically predicted risk of AF was associated with a shorter P-wave duration (*P* = 4 × 10^−4^; **Table I** in **S1 Appendix**). However, the effect size was very small, with an approximately 10% change in AF risk accounting for a 0.1 ms change in P-wave duration, explaining only 1/50th of the causal effect estimate observed for P-wave duration on AF risk described previously (approximately 9% change in AF risk per 5 ms change in P-wave duration).

#### Sensitivity analyses relating to pleiotropy and other assumption violations

Other statistical sensitivity analyses were carried out in UK Biobank to further investigate potential pleiotropic effects of the genetic variants in the ECG scores (**Table J** in **S1 Appendix**). The inverse variance weighted approach (random effects), combining individual causal estimates across the selected genetic variants, gave comparable estimates to the genetic score as anticipated. However, the effects of P-wave duration and QT interval on risk of AF were not statistically significant. The weighted median and weighted mode MR methods gave comparable results to the primary analyses. MR–Egger analyses provided no evidence of directional pleiotropy within either the PR interval or QT interval scores (Egger intercept *P* = 0.69 and *P* = 0.99, respectively) and also showed comparable causal effect estimates for AF to the primary results for both PR interval and QT interval, albeit nonsignificant. For P-wave duration, MR–Egger analyses indicated possible bias (Egger intercept *P* = 0.02), which may be a result of directional pleiotropy, but the bias-adjusted causal effect estimate showed a significant association between P-wave duration and AF (OR: 0.64; 95% CI: 0.45 to 0.89, *P* = 0.009. The results after excluding outlying genetic variants based on the MR-PRESSO method were comparable to the primary results for both PR interval and QT interval. In contrast, after 3 of the 8 genetic variants comprising the P-wave duration score were excluded by the MR-PRESSO method, the association between P-wave duration and AF risk was attenuated and no longer statistically significant. Information from the PhenoScanner database suggested that there were no meaningful effects of the genetic variants contributing to the scores on other cardiovascular phenotypes (**Table K** in **S1 Appendix**). Funnel plots to visualise the causal estimates for each variant within each ECG genetic score are shown in **Figs E–G** in **S1 Appendix**.

## Discussion

AF is characterised by a disruption in coordinated atrial electrical activity. This study uses MR methods to provide novel evidence supporting causal relationships between lifelong differences in cardiac electrical parameters and risks of developing both AF and non-AF SVT. In particular, genetically predicted differences in PR interval showed robust associations across a broad range of analyses and mechanistic pathways.

### ECG parameters as predictors of AF

ECG parameters reflecting the propagation of the electric impulse through the atria to the ventricular myocardium have been associated with incident AF in a number of large observational studies [5–10,36]. However, risk factors for AF, such as age and heart failure, a history of AF, and use of anti-arrhythmic or rate-controlling medications, also influence ECG parameters, limiting any causal inferences from observational data. In the largest observational study to examine associations between atrial ECG parameters and AF [7,9], investigators observed the highest risk of AF in those with abnormally long PR intervals (>200 ms), but these observations were made in patients who had been referred for ECGs by their general practitioner, who therefore may have been at higher risk of cardiovascular disease than the general population. Indeed, risk scores for predicting incident AF use an abnormal PR interval as a component of the score [36,37], again implying that longer atrial conduction time in older patients with cardiovascular disease is a risk indicator for, but not necessarily a causal determinant of, AF.

In UK Biobank, in the subgroup of participants who had measured 12-lead ECG parameters, these mainly fell within normal reference ranges. Assuming similar characteristics across our whole cohort, a longer genetically predicted P-wave duration was associated with a lower risk of AF, consistent with data from observational studies over the same range of values [6,7]. A longer PR interval was also associated with a lower risk of AF but over a range of values (approximately 155 to 175 ms) that had previously been associated with a higher risk of AF in observational studies [9]. The consistent effects of the ECG genetic scores on risk of so-called “lone” AF further support the conclusion that the potential causal associations were independent of any shared links with major AF risk factors. Furthermore, being at higher genetic risk of AF was not associated with altered ECG parameters to a meaningful extent, suggesting that bidirectional relationships do not explain the observed effects of ECG parameters on risk of AF.

In UK Biobank, our results show a nonsignificant association between QT interval and AF, and after meta-analysis, a small association between a longer QT_c_ interval within the normal range (approximately 410 to 430 ms) and a lower risk of AF. Observational studies have typically showed strongest associations with AF once the QT interval was abnormal (i.e., QT_c_ > 450 ms) [13,14]. The QT interval prolongs with age, concomitant cardiovascular disease, or as a result of anti-arrhythmic medication for AF [38]. Mechanistically, it is difficult to link ventricular repolarisation directly to AF, although it is plausible that atrial and ventricular depolarisation (the QRS interval, a small part of the QT) would share similar electrophysiological properties. Overall, an altered QT interval may be more likely to reflect a similarly altered atrial electrical substrate than being directly causal itself.

Previous GWAS for the PR interval reported that some individual genetic variants that affected the PR interval were associated with risk of AF [39,40]. Here, the different variants’ effects on AF were not consistent with their effects on PR interval and were not quantified. The strengths of a formal MR analysis such as ours are that all the genetic variants are included as appropriately weighted surrogates for the exposure of interest, with estimation of the effect of lifelong exposure on the outcome and subsequent consideration of pleiotropy.

### Mechanistic insights into pathophysiology of arrhythmias

The relationship between abnormal prolongation of the P-wave and PR interval and a higher risk of AF has been interpreted as reflecting prolonged atrial conduction times, secondary to structural remodelling and fibrosis, promoting reentry circuits, and favouring the maintenance of AF [41]. We found that, even within their normal range, longer P-wave duration and PR interval were associated with lower risks of both AF and SVT, implying that shorter atrial electrical conduction times may directly increase the risk of both arrhythmias. The mechanism underpinning the causal link between our observed findings may seem less intuitive, but has some supporting evidence in the literature. For example, consistent with our findings, commonly used anti-arrhythmic drugs for AF such as flecainide, propafenone, and amiodarone are known to prolong the PR interval, and yet reduce risk of AF and SVT [42]. Within the ion channel scores, variants in the *SCN5A* gene that lengthened P-wave duration and PR interval were associated with a lower risk of AF and SVT, in keeping with the anti-arrhythmic action of flecainide, which, by blocking Na_v_1.5 (the sodium channel encoded by *SCN5A*), prolongs the PR interval and prevents AF and SVT recurrence. Furthermore, consistent with the overall scores, ion channel scores for atrial conduction parameters affected risk of AF and SVT, whereas ion channel scores for ventricular repolarisation did not, suggesting that chamber specific drug targets may have therapeutic potential. While familial studies investigating “lone” AF due to *SCN5A* have yielded conflicting results regarding pathogenicity [43,44], more recent data suggest that genetic variation in the nearby *SCN10A* regulates *SCN5A* expression [45], with alleles associated with prolonged PR interval reducing *SCN5A* mRNA levels in a dose dependent manner, consistent with our findings.

Both P-wave duration and PR interval are affected by atrial volume and tissue characteristics (e.g., fibrosis, muscle fibre orientation, etc.). In our study, the ECG scores were not associated with differences in left atrial volume, suggesting the genetic scores did not alter the risk of AF through this well-established mechanism.

Large-scale epidemiological studies have reported a “U-shaped relationship” between ECG parameters and AF, with individuals with both short and long intervals at higher risk [9,12,37]. Interestingly, individuals with shorter intervals were much younger and had a very low prevalence of cardiovascular comorbidities compared to those with longer intervals, although both groups had similarly increased risks of AF. In other studies, individuals with a short PR interval but no other signs of preexcitation have been reported to have an increased incidence of supraventricular arrhythmia, including SVT, AF, and atrial flutter [46,47]. Overall, this suggests distinct mechanisms of arrhythmogenicity in the presence or absence of altered atrial structure.

There is limited large-scale epidemiological evidence regarding SVT. Similarly, the heritability of non-AF SVT is not well understood, with most cases thought to be sporadic, although there is some evidence that Wolff-Parkinson-White (WPW) syndrome has a hereditary basis [48]. Patients with WPW syndrome (comprising approximately 10% of SVT cases in our dataset) have a shortened PR interval on ECG and a higher risk of AF that persists despite successful accessory pathway ablation, suggesting the presence of an additional atrial pro-arrhythmic substrate beyond the accessory pathway [49]. More recently, an increased risk of AF has been reported in patients who have undergone ablation for atrioventricular nodal re-entrant tachycardia [50], which is the commonest non-AF form of SVT, comprising approximately 60% of cases and typically due to reentry within dual “slow” and “fast” pathways. Similarly, patients with short PR interval and normal QRS duration, as originally described by Lown and colleagues [47], present with a variety of atrial arrhythmias in the absence of a defined accessory pathway, suggesting that fast impulse transmission through the atria and to the ventricles may be arrhythmogenic per se.

Overall, our study provides evidence supporting the concept that AF may be a common arrhythmia representing different mechanisms and atrial substrates. Interestingly, our specific findings seem to be also applicable to non-AF SVT, which conventionally has not been thought to be similar to AF mechanistically [17]. This suggests that more individualised ascertainment and therapeutic targeting of specific mechanisms of arrhythmia is warranted.

### Limitations

The GWAS for ECG parameters that were used for genetic variant selection were conducted in participants with European ancestry, and UK Biobank chiefly includes white British individuals; consequently, our analyses are restricted to this population. Further studies are needed to assess the generalisability of our results across ethnicities. Furthermore, while UK Biobank includes comprehensive phenotyping data, information from AF diagnoses recorded in primary care was not included in the present study due to limited availability.

P-wave duration is part of the PR interval, and it is therefore difficult to robustly assess its relevance alone. Further, the P-wave duration genetic risk score was comprised of 8 variants with the most heterogeneity in terms of effects of individual SNPs and greater variability across results from the sensitivity analyses when compared with the PR interval genetic risk score. Limited analyses suggest that the effects of PR interval are unaffected by P-wave duration, and so it is likely that components of both atrial depolarisation and atrioventricular nodal conduction time affect the risk of AF and SVT. Consequently, we refer to atrial electrical conduction times to encompass the underlying biological mechanism behind both P-wave duration and PR interval. It is also not clear whether the weaker effects of QT interval on AF reflect a small but genuine impact of ventricular characteristics on AF or some common heritability between atrial and ventricular electrical traits.

A potential limitation of any MR study is pleiotropy—where the genetic variants affect the outcome (i.e., AF) by a biological pathway not mediated by the risk factor of interest (i.e., the ECG parameters). Multiple sensitivity analyses provided similar effect estimates as the primary analyses, with particularly robust results for PR interval, making it unlikely that pleiotropy had a meaningful impact on the results. The consistent associations of the ECG scores with AF and “lone” AF, and the similar results obtained from the ion channel scores, provide additional evidence of the robustness of the reported associations.

The effect sizes on AF and SVT observed in this study represent a lifelong cumulative effect of differences in ECG parameters and are not necessarily directly comparable to those derived from observational or clinical studies. Additionally, the study results may be limited to the normal reference ranges of the ECG measurements, as only these ranges were observed in a subgroup of UK Biobank participants. As with any large middle-aged cohort, UK Biobank includes participants with a wide range of medical conditions who may receive various medications that affect measured ECG parameters and thus influence the range of values represented in this study. Our findings do not rule out different relationships between the ECG parameters and AF outside of the ranges studied, and thus further studies exploring wider ECG ranges and nonlinear relationships with AF are warranted.

## Conclusions

We have provided evidence that supports, within the normal range of ECG parameters, a causal relationship between lifelong differences that reflect longer atrial electrical conduction and lower risks of AF and SVT. Our study also provides mechanistic insight into the underlying pathophysiology of these arrhythmias and shows that the effects of ECG parameters on AF appear to be driven partly by differences in atrial electrical substrate, and not atrial size or major cardiovascular comorbidities. This emphasises that substantial variation in the mechanisms underpinning AF exists and warrants the consideration of more individualised treatment strategies.

## Supporting information

S1 Checklist STROBE Statement—checklist of items. STROBE, Strengthening the Reporting of Observational Studies in Epidemiology.
(DOC)

S1 Appendix Supporting methods, figures and tables. (PDF)

## Abbreviations

AF: atrial fibrillation
CI: confidence interval
ECG: electrocardiographic
GWAS: genome-wide association study
ICD-10: International Statistical Classification of Diseases-10th Revision
MR: mendelian randomization
MRI: magnetic resonance imaging
OR: odds ratio
STROBE: Strengthening the Reporting of Observational Studies in Epidemiology
SVT: supraventricular tachycardia
WPW: Wolff-Parkinson-White
