# Supplementary material for: Assessment of the causal relevance of ECG parameters for risk of atrial fibrillation: A mendelian randomisation study
Source: PLoS Med. 2021 May 13;18(5):e1003572. doi: 10.1371/journal.pmed.1003572 (PMC8118296; doi:10.1371/journal.pmed.1003572)
Supplement: S1 Appendix — (PDF) [file pmed.1003572.s002.pdf]

S1 APPENDIX

Supporting methods, figures and tables

SUPPORTING METHODS 1
Data sources 1
Selection of genetic variants for P-wave duration score 1
Selection of genetic variants for PR interval duration score 1
Selection of genetic variants for QT interval score 2
Selection of genetic variants for ion channel scores 2
Explained variance and power calculations 2
Sensitivity Analyses 3
Qualitative evaluation of pleiotropic effects 3
Genetically determined risk of atrial fibrillation and effect on 12-lead ECG parameters 3
Software 3
SUPPORTING FIGURES 4
Fig A. Generation of main analyses datasets 5
Fig B. Genetic variant selection details 6
Fig C. Distribution of genetic scores in the UK Biobank population 7
Fig D. Effects of genetically determined ECG parameters on risk of 'lone' atrial fibrillation in UK Biobank 8
Fig E. Funnel plot for SNPs in P-wave duration score 9
Fig F. Funnel plot for SNPs in PR interval score 10
Fig G. Funnel plot for SNPs in QT interval score 11
SUPPORTING TABLES 12
Table A. Phenotype definition of atrial fibrillation in UK Biobank 13
Table B. Phenotype definitions of other diseases in UK Biobank 14
Table C. SNPs included in P-wave duration score 15
Table D. SNPs included in PR interval score 16
Table E. SNPs included in QT interval score 18
Table F. Power calculations for genetic ECG scores 20
Table G. SNPs included in atrial fibrillation score 21
Table H. Baseline characteristics of participants with supraventricular tachycardias in UK Biobank 25
Table I. Effect of atrial fibrillation genetic risk score on 12-lead ECG parameters 26
Table J. Sensitivity analyses for genetically predicted effects of ECG parameters on atrial fibrillation in UK Biobank 27
Table K. Phenoscanner derived details for non-ECG parameter associated traits for SNPs in ECG scores 28
SUPPORTING REFERENCES 30

SUPPORTING METHODS

Data sources

For participants in England, data from in-patient hospital admissions was obtained from Hospital Episode Statistics for England (NHS digital) from 1996 – 31st March 2017, with information on cause of death from NHS Digital from April 2006 – 31st January 2018. For participants in Wales, data from in-patient hospital admissions was obtained from the Patient Episode Database for Wales (Secure Anonymised Information Linkage, Wales) from 1999 – 29th February 2016, with information on cause of death from NHS Digital from April 2006 – 31st January 2018. For participants in Scotland, data from in-patient hospital admissions was obtained from the Scottish Morbidity Record (Information and Statistics Division, Scotland) from 1996 – 31st October 2016 with information on cause of death provided by the same organisation from April 2006 -30th November 2016. See http://biobank.ctsu.ox.ac.uk/crystal/exinfo.cgi?src=Data_providers_and_dates for further details.

Selection of genetic variants for P-wave duration score

A large-scale genome-wide association study (GWAS) meta-analysis for P-wave duration identified 9 individual single nucleotide polymorphisms (SNPs) that met genome-wide significance in nearly 38,000 individuals of European ancestry from a variety of studies, but not including UK Biobank [1]. Briefly, all studies used in the GWAS meta-analysis excluded participants with AF, an implanted pacemaker, Wolff-Parkinson-White syndrome, complete heart block and those who received medications altering atrioventricular nodal conduction (β-blockers, dihydropyridine calcium channel blockers, Vaughan-Williams class I and III anti-arrhythmic medications and digoxin) as well as those in whom the P-wave duration could not be determined. In each cohort, P-wave duration was adjusted in a linear model for age, sex, RR interval and principal components of ancestry.

Taking the summary data from the meta-analysis, further clumping of SNPs was performed using linkage disequilibrium (LD) score of $r^2<0.01$ within a ±250kb window using 1000 Genomes Phase 1 (EUR) reference data. One of the SNPs identified (rs148020424) was flagged as an insertion-deletion and was excluded from our score leaving 8 SNPs (**Table C**) that were included in our P-wave duration score.

Selection of genetic variants for PR interval duration score

A large-scale GWAS meta-analysis for PR interval duration identified 61 individual SNPs that met genome-wide significance in over 92,000 individuals of European ancestry from a variety of studies, but not including UK Biobank [2]. Briefly, all studies used in the GWAS meta-analysis excluded participants with AF, history of myocardial infarction or heart failure, extreme PR interval values (≤80ms or ≥320ms), Wolff-Parkinson-White syndrome, pacemaker implantation and use of β-blockers, Vaughan-Williams class I and III anti-arrhythmic medications or digoxin. In each cohort, age, sex, height, body mass index and principal components of genetic ancestry were included as covariates.

Taking the summary data from the meta-analysis, further clumping of SNPs was performed using LD score of $r^2<0.01$ within a ±250kb window using 1000 Genomes Phase 1 (EUR) reference data. This yielded 52 SNPs (**Table D**) that were included in our PR interval score.

Selection of genetic variants for QT interval score
A large-scale GWAS meta-analysis for QT interval included ~103,000 individuals of European ancestry from a variety of studies, but not including UK Biobank [3]. Additionally, an exome-chip sequencing GWAS was published including 95,626 individuals [4]. All studies used in both of the meta-analyses excluded participants with AF, QRS duration >120ms, bundle branch block, and, when available, electronic pacemaker use or QT-altering medication use. In each cohort, QT interval duration was adjusted for age, sex, RR interval and principal components of genetic ancestry and tested for association with 2.5million directly genotyped or imputed SNPs.

Taking the summary data from the meta-analysis and restricting to variants meeting GWAS significance, further clumping of SNPs was performed using LD score of $r^2<0.01$ within a ±250kb window using 1000 Genomes Phase 1 (EUR) reference data. This yielded 54 SNPs (**Table E**) that were included in our QT interval score.

Selection of genetic variants for ion channel scores
To test biological plausibility of the ECG scores, scores with genetic variants mapping to specific biological pathways involving electrical ion channels only were generated. To identify these in an unbiased way, the SNPnexus tool [5] was used to annotate the variants to known gene regions. Each variant in the main analysis was annotated to the closest gene within 25kb. Biological pathways were investigated using the Gene Ontology (GO) tool [6,7].Variants that were annotated to genes involved in known biological pathways affecting action potential duration were included in the ion channel scores for each ECG parameter.

Explained variance and power calculations
The strength of the genetic variants used was assessed by calculation of the explained variance (R^2) of the trait using the following formula described elsewhere [8].

$$R^2 = \sum R_i^2 = \frac{\beta_i^2 \times var(SNP_i)}{var(X)}$$

where

$$var(SNP_i) = 2 \times MAF_{SNP_i} \times (1 - MAF_{SNP_i})$$

The total variance explained by the score is the sum of the individual variances explained by *i* SNPs within the score. β_i is the estimated effect of the ith SNP on the ECG parameter; $var(SNP_i)$ is calculated using the minor allele frequencies (MAF) of each SNP and $var(X)$ is the variance of the ECG parameter. In this case $var(X)$ was set as 1 standard deviation (SD) unit, from the reported SDs of the original population phenotypes in the GWAS.

To assess the potential for weak instrument bias, *F*-statistics for each SNP were calculated as β^2/SE^2 as described elsewhere [9].

We used the online tool https://sb452.shinyapps.io/power/ for power calculations for Mendelian Randomisation analyses. See **Table F** for further details.

Sensitivity Analyses

Qualitative evaluation of pleiotropic effects

For variants contained in each score, we used PhenoScanner (http://www.phenoscanner.medschl.cam.ac.uk/information.html) [10] to extract phenotypes with a screening *P*-value < 0.001 and using proxies for any of the variants in the scores with r^2 > 0.8 in 1000 Genomes Phase 3 data. We then limited our analysis to reports of GWAS significant *P*-values (< 5 × 10^{-8}) only. We selected the proxy with the highest r^2 with the variant in the score for each individual trait described. In the case of multiple reports of the same trait, we selected the study with the strongest association described to report (i.e. smallest *P*-value). We examined these for consistent associations with known confounders.

Genetically determined risk of atrial fibrillation and effect on 12-lead ECG parameters

To generate an AF genetic risk score for the bi-directional analysis, we used the latest large-scale GWAS for AF including 60,620 cases of AF and 970,216 controls of predominantly European ancestry from a variety of studies [11]. As the discovery GWAS included UK Biobank as constituent study, we used weights for the individual SNPs published in the GWAS for the SNPs derived from studies with UK Biobank excluded. Data with UK Biobank excluded weights was available for 142 SNPs reaching GWAS significance (*P* < 5 × 10^{-8}).

Taking the summary data from the meta-analysis, further clumping of SNPs was performed using LD score of r^2<0.01 within a ±250kb window using 1000 Genomes Phase 1 (EUR) reference data. This yielded 133 SNPs (**Table G**) that were included in the AF risk score.

In UK Biobank, genetic scores were calculated for each participant by summing the number of AF risk increasing alleles of each SNP weighted by their effect size on the AF risk (unit ln odds). Effects of the AF risk score on 12-lead ECG parameters were estimated using linear regression adjusted for sex, genotyping array and forty principal components of ancestry (as provided by UK Biobank), with effects reported per unit ln odds (~2.7 OR increase).

Software

We used the R package Mendelian Randomization (v0.4.0 - https://cran.r-project.org/web/packages/MendelianRandomization/index.html) to generate inverse variance weighted estimates, weighted median estimates and MR-Egger estimates. The weighted mode based estimate function was obtained from GitHub (https://github.com/MRCIEU/mbe). The MR-PRESSO R package (https://github.com/rondolab/MR-PRESSO) was used to exclude outlying genetic variants as described previously [12]. The R package metafor (v2.0.0 - http://www.metafor-project.org/doku.php) was used for meta-analysis.

SUPPORTING FIGURES

Fig A. Generation of main analyses datasets

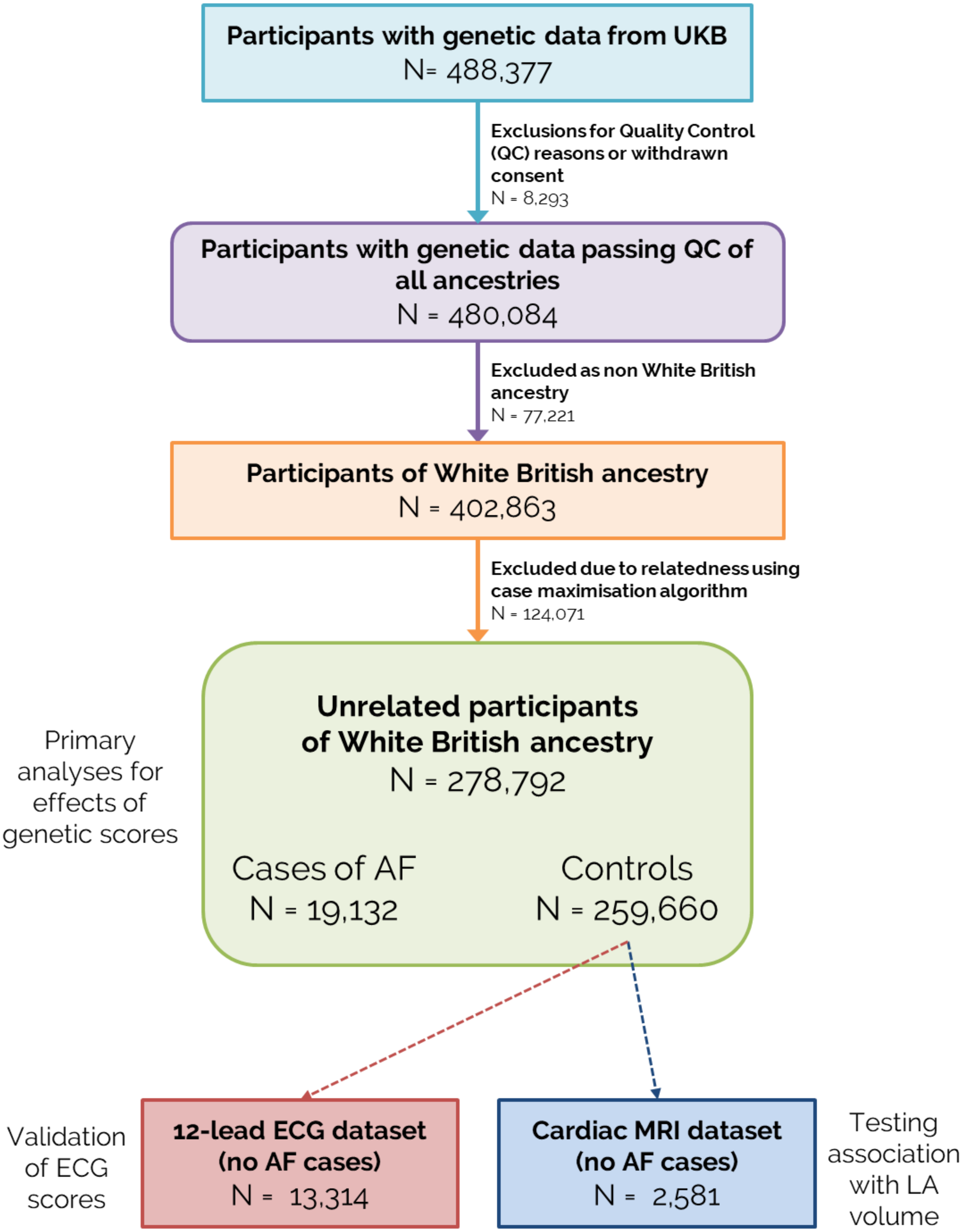

AF: Atrial fibrillation; ECG: Electrocardiogram; LA: Left atrium; MRI: Magnetic resonance imaging; SVT: Supraventricular tachycardia; UKB: UK Biobank

Fig B. Genetic variant selection details

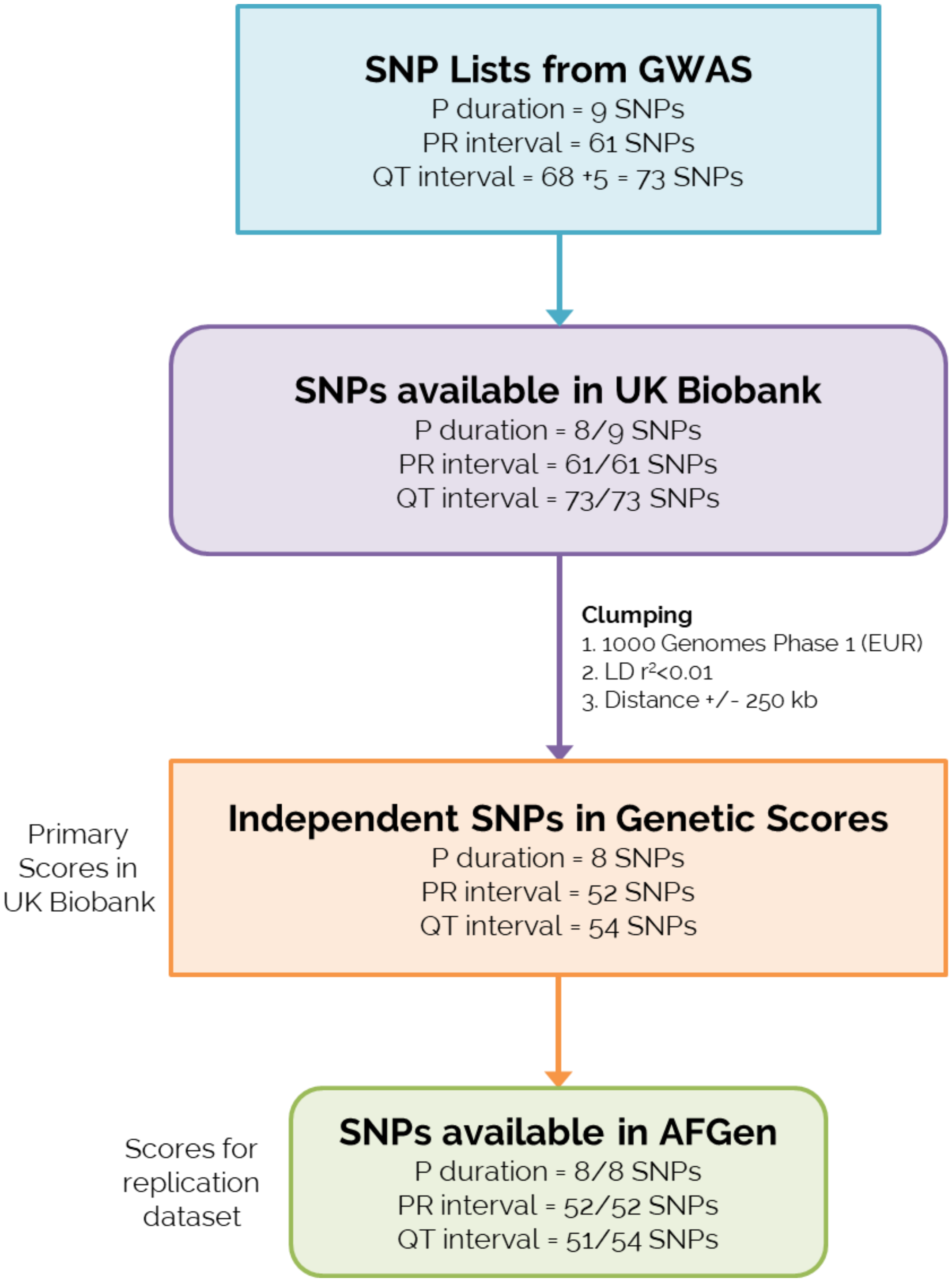

AF: Atrial fibrillation; GWAS: Genome wide association study; LD: Linkage disequilibrium; SNP: Single nucleotide polymorphism

Fig C. Distribution of genetic scores in the UK Biobank population

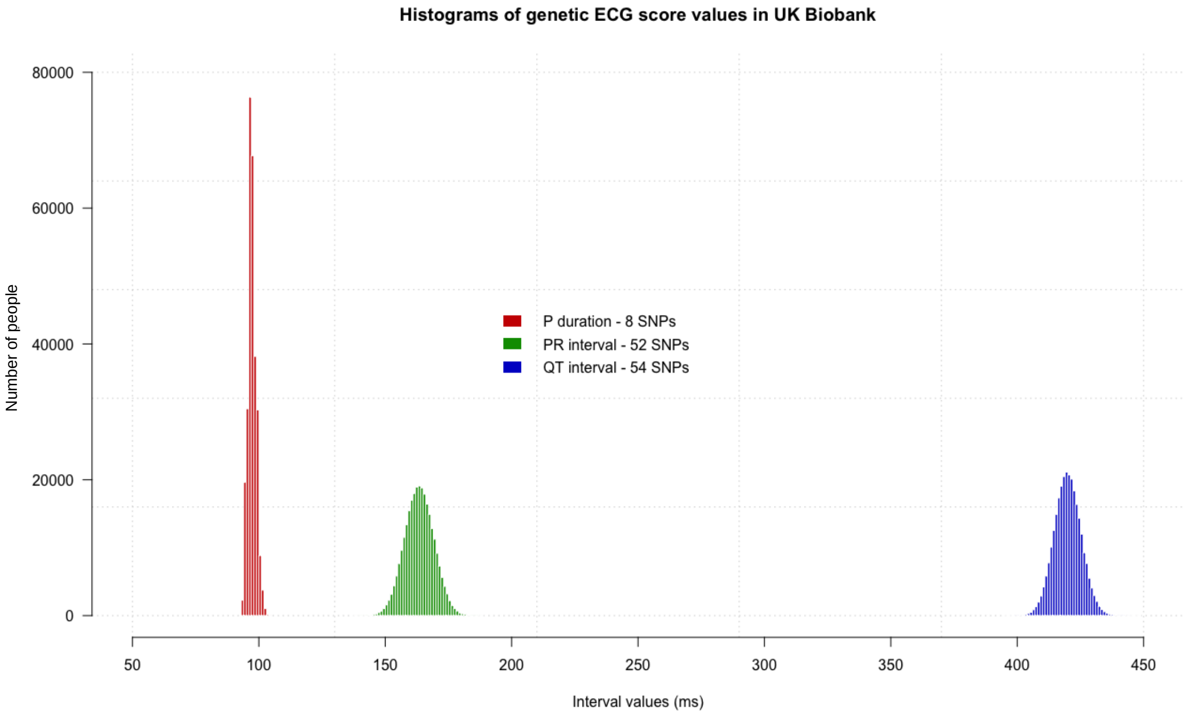

Weighted genetically predicted ECG score values.

Total number of people in each score is 278,792.

Mean ± standard deviation of scores:
P duration: 97.3 ± 1.6 ms
PR interval: 163.4 ± 5.8 ms
QT interval: 420.0 ± 5.3 ms

Fig D. Effects of genetically determined ECG parameters on risk of 'lone' atrial fibrillation in UK Biobank

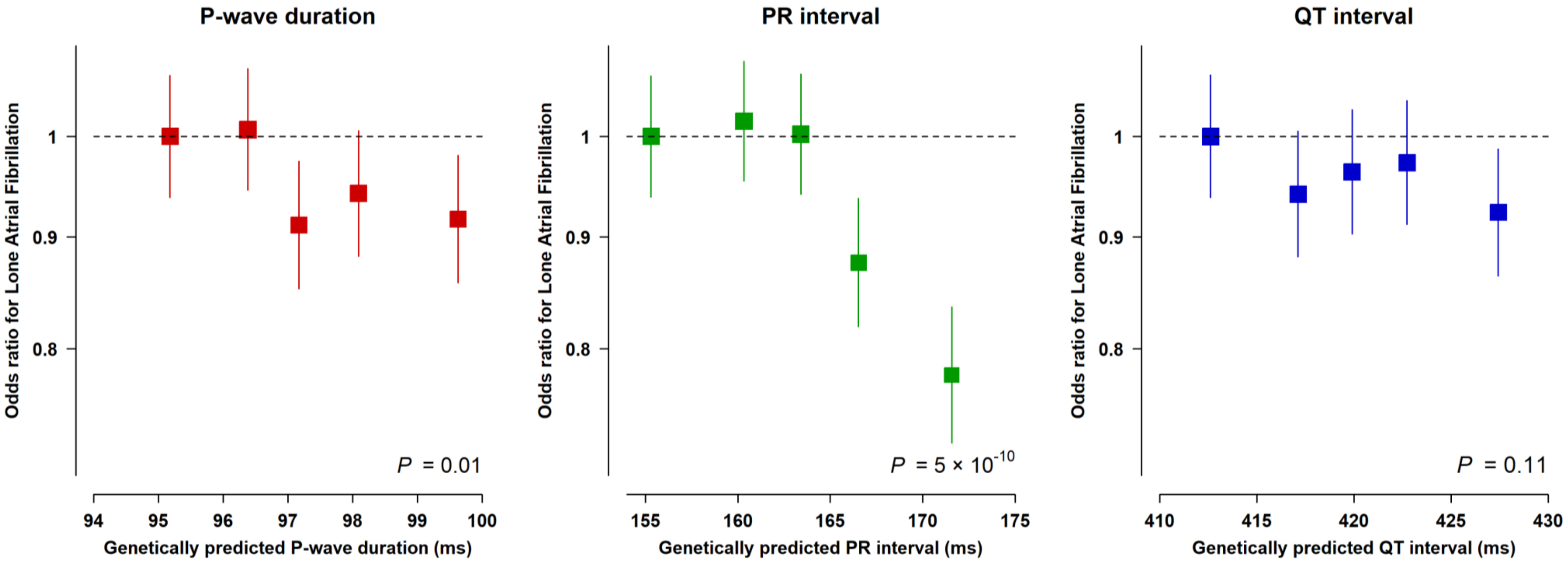

Odds ratios (OR) for 'lone' atrial fibrillation per quintile of genetic score (defined in non-AF cases) in 278,792 participants in UK Biobank. Boxes represent effect estimates with their size inversely proportional to variance. Solid lines represent 95% confidence intervals (CI) calculated using floating absolute risks. ORs are adjusted for genotyping array, sex and forty principal components of ancestry. *P* calculated across continuous genetic score values adjusting for sex, genotyping array and forty principal components of ancestry.

Fig E. Funnel plot for SNPs in P-wave duration score

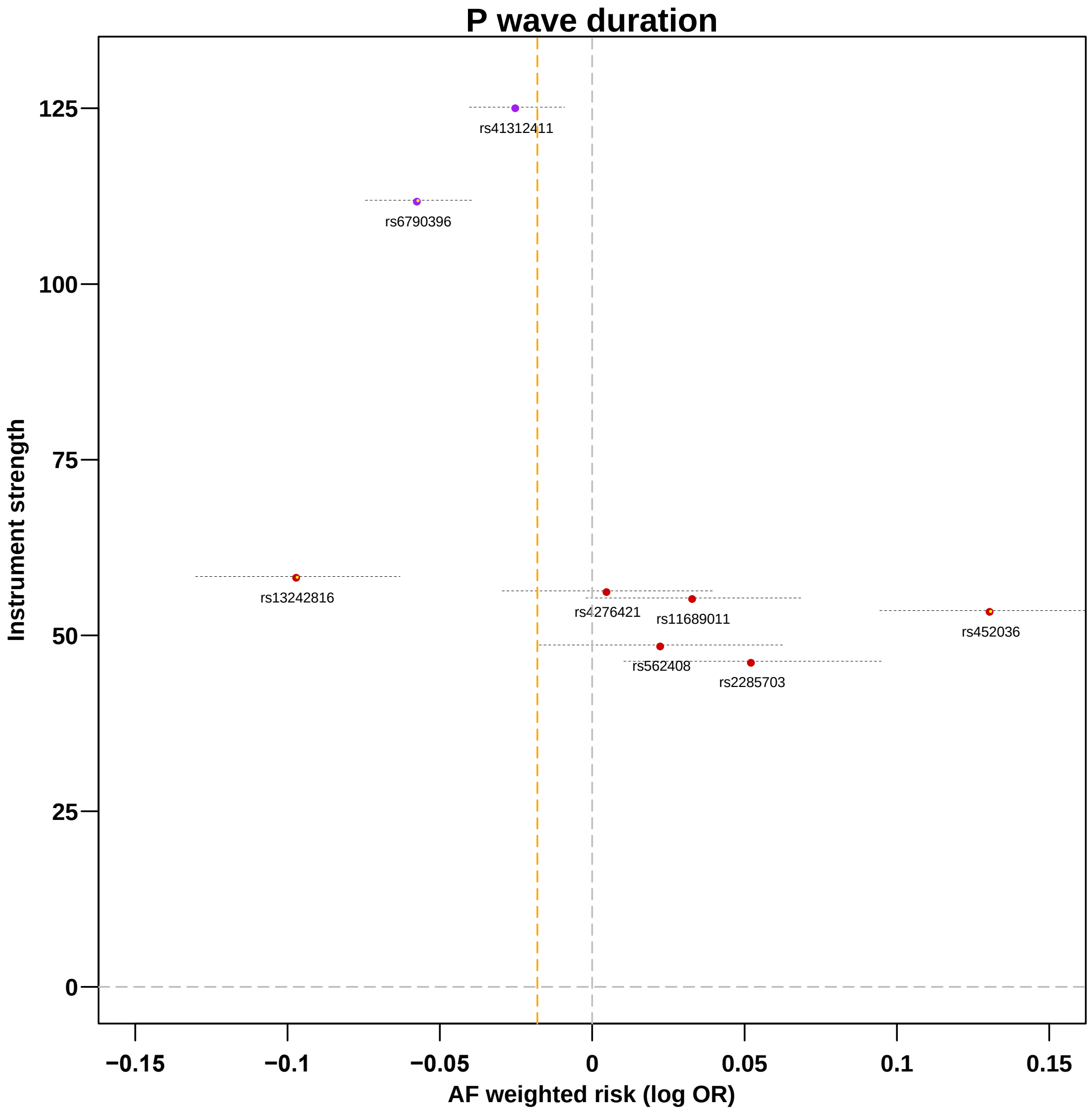

Funnel plot showing individual single nucleotide polymorphism (SNP) effects on atrial fibrillation (AF) expressed as log odds ratio (OR) estimates from UK Biobank data vs. strength of SNP in genetic P-wave duration score (effect on P-wave duration / standard error of estimate of effect on AF). Orange dotted line is the overall effect estimate for the score, red circles represent SNPs in P-wave duration score, purple circles represent SNPs in ion channel sub-score, inner yellow circles represent a SNP excluded by MR-PRESSO method.

Fig F. Funnel plot for SNPs in PR interval score

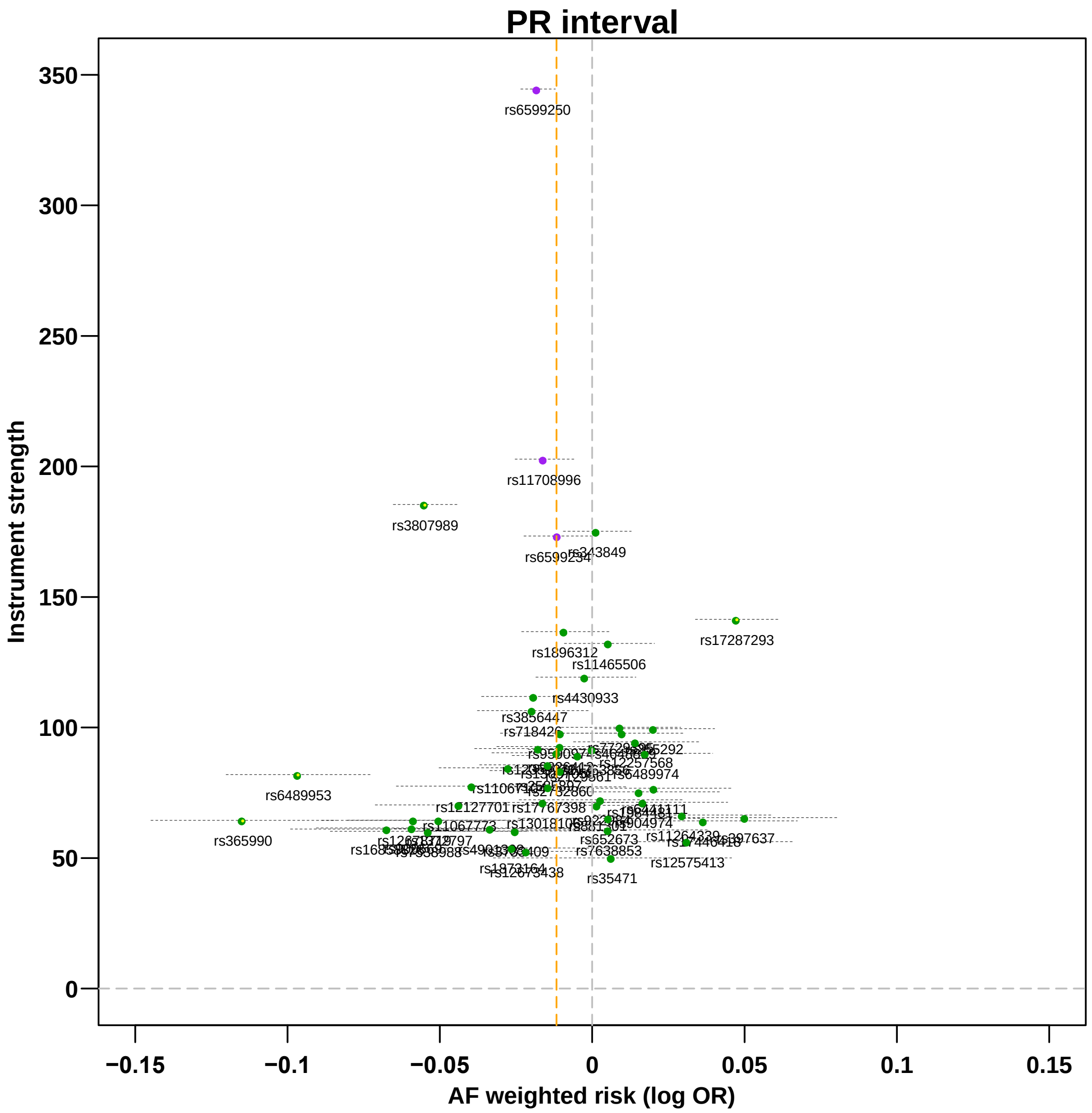

Funnel plot showing individual single nucleotide polymorphism (SNP) effects on atrial fibrillation (AF) expressed as log odds ratio (OR) estimates from UK Biobank data vs. strength of SNP in genetic PR interval score (effect on PR interval / standard error of estimate of effect on AF). Orange dotted line is the overall effect estimate for the score, green circles represent SNPs in PR interval score, purple circles represent SNPs in ion channel sub-score, inner yellow circles represent a SNP excluded by MR-PRESSO method.

Fig G. Funnel plot for SNPs in QT interval score

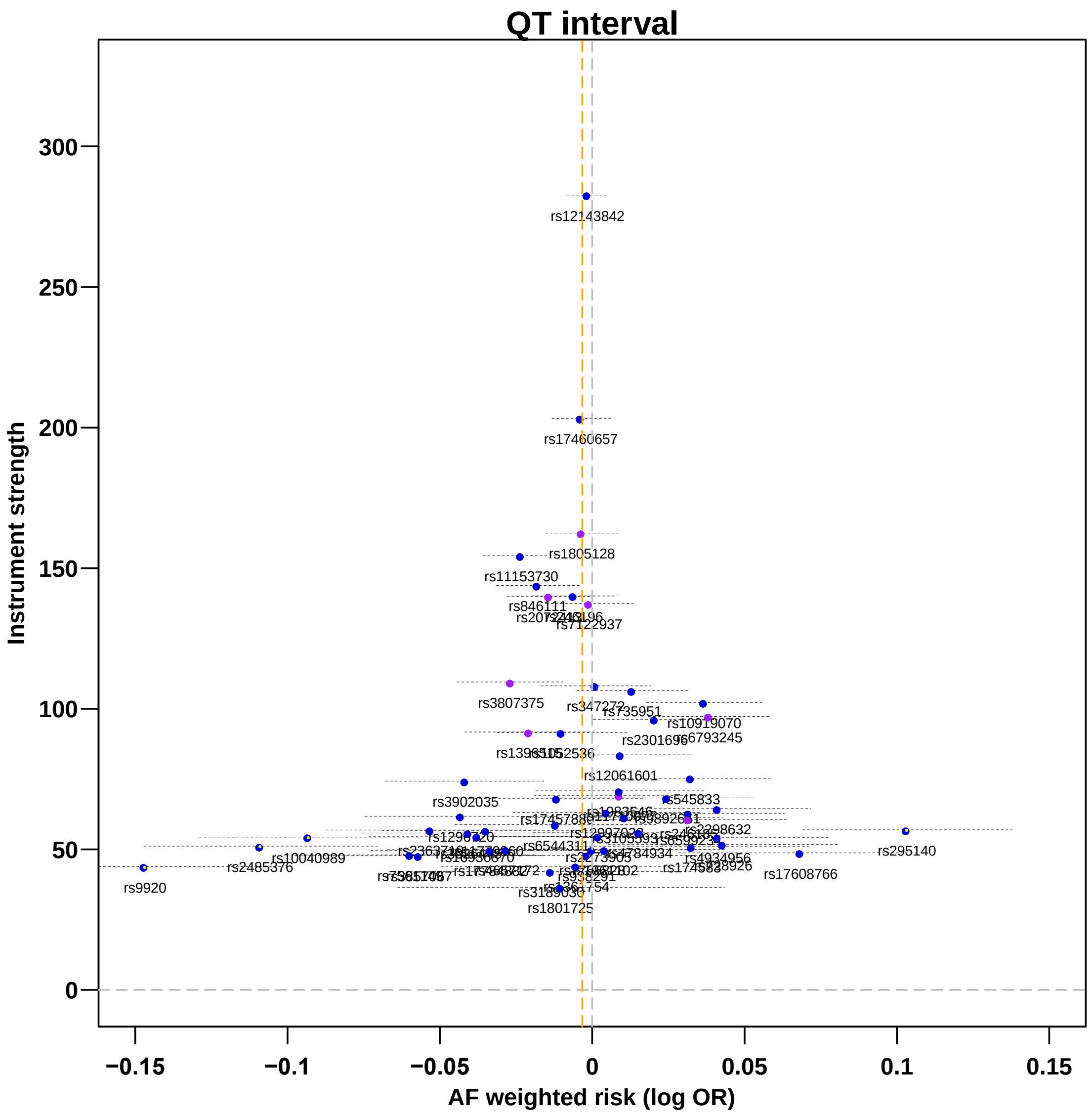

Funnel plot showing individual single nucleotide polymorphism (SNP) effects on atrial fibrillation (AF) expressed as log odds ratio (OR) estimates from UK Biobank data vs. strength of SNP in genetic QT interval score (effect on QT interval / standard error of estimate of effect on AF). Orange dotted line is the overall effect estimate for the score, blue circles represent SNPs in QT interval score, purple circles represent SNPs in ion channel sub-score, inner yellow circles represent a SNP excluded by MR-PRESSO method.

SUPPORTING TABLES

Table A. Phenotype definition of atrial fibrillation in UK Biobank

Field	Description	Algorithm elements
20002	Non-cancer illness code, self-reported	Atrial Fibrillation – 1471, Atrial Flutter – 1483
20004	Operation code	Cardiac Ablation – 1553, Cardioversion – 1524
41202	Diagnoses – main ICD10	Atrial Fibrillation or Flutter – I48*
41204	Diagnoses – secondary ICD10	Atrial Fibrillation or Flutter – I48*
41200	Operative procedures – main OPCS	K22.3 Exclusion of left atrial appendage,<br>K57.1 Percutaneous transluminal ablation of atrioventricular node,<br>K57.5 Percutaneous transluminal ablation of atrial wall,<br>K62.1 Percutaneous transluminal ablation of pulmonary vein to left atrium conducting system,<br>K62.2 Percutaneous transluminal ablation of atrial wall for atrial flutter,<br>K62.3 Percutaneous transluminal ablation of conducting system of heart for atrial flutter,<br>K62.4 Percutaneous transluminal internal cardioversion,<br>K62.5 Percutaneous transluminal occlusion of left atrial appendage,<br>X50.1 Direct current cardioversion,<br>X50.2 External cardioversion
41210	Operative procedures – secondary OPCS	K22.3 Exclusion of left atrial appendage,<br>K57.1 Percutaneous transluminal ablation of atrioventricular node,<br>K57.5 Percutaneous transluminal ablation of atrial wall,<br>K62.1 Percutaneous transluminal ablation of pulmonary vein to left atrium conducting system,<br>K62.2 Percutaneous transluminal ablation of atrial wall for atrial flutter,<br>K62.3 Percutaneous transluminal ablation of conducting system of heart for atrial flutter,<br>K62.4 Percutaneous transluminal internal cardioversion,<br>K62.5 Percutaneous transluminal occlusion of left atrial appendage,<br>X50.1 Direct current cardioversion,<br>X50.2 External cardioversion
40001	Underlying (primary) cause of death: ICD10	Atrial Fibrillation or Flutter – I48*
40002	Contributory (secondary) causes of death: ICD10	Atrial Fibrillation or Flutter – I48*

*****Indicates all ICD10 sub-codes under main heading included

Table B. Phenotype definitions of other diseases in UK Biobank

Disease	Code type	Codes and description
Supraventricular tachycardia	ICD10	Preexcitation syndrome – I45.6
		Supraventricular tachycardia – I47.1
		Atrial premature depolarisation – I49.1
	OPCS	Open division of accessory pathway within heart – K52.4
		Percutaneous transluminal ablation of conducting system of heart NEC – K57.2
		Percutaneous transluminal ablation of accessory pathway – K57.4
	Self-reported	Illness codes – 1484, 1487
Coronary Heart Disease	ICD10	Angina – I20*
		Acute Myocardial Infarction – I21*
		Subsequent Myocardial Infarction – I22*
		Certain current complications following acute myocardial infarction – I23*
		Other acute ischaemic heart diseases – I24*
		Chronic ischaemic heart disease – I25*
	OPCS	Transluminal balloon angioplasty of coronary artery – K49*
		Other therapeutic transluminal operations on coronary artery – K50*
		Percutaneous transluminal balloon angioplasty and insertion of stent into coronary artery – K75*
		Saphenous vein graft replacement of coronary artery – K40*
		Other autograft replacement of coronary artery – K41*
		Allograft replacement of coronary artery – K42*
		Prosthetic replacement of coronary artery – K43*
		Other replacement of coronary artery – K44*
		Connection of thoracic artery to coronary artery – K45*
		Other bypass of coronary artery – K46*
	Self-reported	Illness codes - 1074, 1075
		Operation codes – 1070, 1071, 1095
Heart failure	ICD10	Heart Failure – I50*
		Hypertensive heart disease – I11.0
		Hypertensive heart and renal disease – I13.0, I13.2
	Self-reported	Illness code – 1076
Hypertension	ICD10	Essential (primary) hypertension – I10*
		Hypertensive heart disease – I11*
		Hypertensive heart and renal disease – I13*
		Secondary hypertension – I15*
	Self-reported	Illness codes – 1065, 1072
Diabetes	ICD10	Insulin-dependent diabetes mellitus – E10*
		Non-insulin-dependent diabetes mellitus – E11*
		Other specified diabetes mellitus – E13*
		Unspecified diabetes mellitus – E14*
	Self-reported	Probable Type 1 and Type 2 Diabetes used from algorithm validated in UK Biobank data from Eastwood *et. al*, 2016. [13]

*****Indicates all ICD10 sub-codes under main heading included

Table C. SNPs included in P-wave duration score

SNP (rsID)	Chromosome	Nearest gene locus	Coded allele	Non-coded allele	Weighting for score (ms)
rs562408	1	*SSBP3*	G	A	0.53
rs11689011	2	*EPAS1*	T	C	0.60
rs41312411*	3	*SCN5A*	G	C	1.91
rs6790396*†	3	*SCN10A*	C	G	1.22
rs2285703	4	*CAMK2D*	G	A	0.56
rs4276421	5	*HCN1*	C	T	0.61
rs13242816†	7	*CAV1/CAV2*	T	C	1.21
rs452036†	14	*MYH6*	A	G	0.59

SNP: Single nucleotide polymorphism
*SNPs included in Ion Channel Score
†SNPs excluded by MR-PRESSO method
Weighting for score is per coded allele and reported unaltered from the GWAS [1]

Table D. SNPs included in PR interval score

SNP (rsID)	Chromosome	Nearest gene locus	Coded allele	Non-coded allele	Weighting for score (ms)
rs11264339	1	*KRTCAP2*	C	T	0.709
rs12127701	1	*MYBPHL*	G	A	1.668
rs397637	1	*OBSCN*	T	G	0.769
rs4648819	1	*SKI*	A	G	1.735
rs7538988	1	*EPS15*	T	C	2.129
rs13018106	2	*FIGN*	G	C	0.776
rs1873164	2	*CCDC141*	A	G	0.723
rs2732860	2	*TMEM182*	A	G	0.893
rs3856447	2	*ID2*	A	G	1.218
rs4430933	2	*MEIS1*	A	G	1.299
rs922984	2	*TTN*	T	C	1.518
rs11708996*	3	*SCN5A*	C	G	3.089
rs13087058	3	*PDZRN3*	T	C	0.982
rs16858828	3	*PHLDB2*	C	A	0.853
rs6441111	3	*CCNL1*	C	T	0.817
rs6599234*	3	*SCN10A*	T	A	2.000
rs6599250*	3	*SCN10A*	T	C	3.765
rs7638853	3	*SENP2*	G	A	0.685
rs900669	3	*FRMD4B*	A	T	0.770
rs9826413	3	*EOMES*	T	A	2.024
rs17446418	4	*CAMK2D*	G	T	0.781
rs343849	4	*ARHGAP24*	T	A	2.074
rs3733409	4	*FAT1*	T	C	0.949
rs255292	5	*BNIP1 / NKX2-5*	A	C	1.083
rs7729395	5	*PAM*	T	C	2.361
rs11763856	7	*HERPUD2*	T	C	3.062
rs12673438	7	*TBX20*	G	T	1.360
rs2129561	7	*MKLN1*	G	A	0.973
rs3807989†	7	*CAV1*	A	G	2.035
rs12678719	8	*ZFPM2*	G	C	0.799
rs881301	8	*FGFR1*	C	T	0.757
rs12257568	10	*SH3PXD2A*	T	C	1.036
rs12359272	10	*ALDH18A1*	A	G	1.019
rs12575413	11	*NAV2*	A	G	0.743
rs1372797	11	*NAV2*	G	T	1.067
rs652673	11	*WNT11*	T	C	0.827
rs11067104	12	*TBX5*	G	C	1.381
rs11067773	12	*MED13L*	T	C	1.297

SNP (rsID)	Chromosome	Nearest gene locus	Coded allele	Non-coded allele	Weighting for score (ms)
rs17287293†	12	*C12orf67*	A	G	2.179
rs1896312	12	*TBX3*	C	T	1.604
rs35471	12	*TBX3*	G	A	0.686
rs6489953†	12	*TBX5*	C	T	1.227
rs6489974	12	*TBX3*	A	G	0.973
rs2585897	13	*XPO4*	A	G	1.222
rs718426	13	*EFHA1*	A	G	1.158
rs9590974	13	*LRCH1*	C	A	1.097
rs11465506	14	*IL25*	G	A	6.433
rs17767398	14	*SNORD56B*	G	C	0.951
rs365990†	14	*MYH6*	A	G	0.705
rs4901308	14	*FERMT2*	C	T	0.835
rs904974	15	*TLE3*	T	C	1.062
rs1984481	17	*MYOCD*	G	C	0.810

SNP: Single nucleotide polymorphism
*SNPs included in Ion Channel Score
†SNPs excluded by MR-PRESSO method
Weighting for score is per coded allele and reported unaltered from the GWAS [2]

Table E. SNPs included in QT interval score

SNP (rsID)	Chromosome	Nearest gene locus	Coded allele	Non-coded allele	Weighting for score (ms)
rs10919070	1	*ATP1B1*	A	C	1.68
rs12061601	1	*ATP1B1*	T	C	1.41
rs12143842	1	*NOS1AP*	T	C	3.50
rs1361754	1	*PM20D1*	G	A	0.47
rs17457880	1	*NOS1AP*	G	A	1.90
rs17460657	1	*NOS1AP*	A	C	4.60
rs1983546	1	*ATP1B1*	A	G	0.81
rs2298632	1	*TCEA3*	T	C	0.70
rs347272	1	*NOS1AP*	A	G	1.80
rs4657172	1	*NOS1AP*	G	C	0.81
rs545833	1	*ATP1B1*	T	C	0.90
rs846111	1	*RNF207*	C	G	1.73
rs12997023	2	*SLC8A1*	T	C	1.69
rs295140†	2	*SPATS2L*	T	C	0.61
rs6544311	2	*SLC8A1*	A	C	0.65
rs7561149	2	*TTN-CCDC141*	T	C	0.52
rs938291	2	*SP3*	G	C	0.53
rs11710077*	3	*SCN5A-SCN10A*	T	A	0.92
rs17784882	3	*C3ORF75*	C	A	0.54
rs1801725	3	*CASR*	G	T	0.58
rs6599234*	3	*SCN5A-SCN10A*	A	T	0.70
rs6793245*	3	*SCN5A-SCN10A*	G	A	1.12
rs2363719	4	*SLC4A4*	A	G	0.97
rs3857067	4	*SMARCAD1*	T	A	0.51
rs10040989†	5	*GFRA3*	G	A	0.85
rs11153730	6	*SLC35F1-PLN*	C	T	1.65
rs3902035	6	*SLC35F1-PLN*	T	C	0.85
rs7765828	6	*GMPR*	G	C	0.55
rs2072413*	7	*KCNH2*	C	T	1.68
rs3807375*	7	*KCNH2*	T	C	1.22
rs9920†	7	*CAV1*	C	T	0.79
rs11779860	8	*LAPTM4B*	T	C	0.61
rs16936870	8	*NCOA2*	A	T	0.99
rs1961102	8	*AZIN1*	T	C	0.57
rs2485376†	10	*GBF1*	G	A	0.56
rs3189030	10	*NRAP*	G	A	0.48
rs4934956	10	*ZNF37A*	T	C	0.58

SNP (rsID)	Chromosome	Nearest gene locus	Coded allele	Non-coded allele	Weighting for score (ms)
rs174583	11	*FADS2*	C	T	0.57
rs2301696*	11	*KCNQ1*	C	G	1.14
rs7122937*	11	*KCNQ1*	T	C	1.93
rs3026445	12	*ATP2A2*	C	T	0.62
rs728926	13	*KLF12*	T	C	0.57
rs2273905	14	*ANKRD9*	T	C	0.61
rs3105593	15	*USP50-TRPM7*	T	C	0.66
rs1296720	16	*CREBBP*	C	A	0.83
rs246185	16	*MKL2*	C	T	0.72
rs246196	16	*CNOT1*	T	C	1.73
rs4784934	16	*CNOT1*	A	G	0.67
rs735951	16	*LITAF*	G	A	1.15
rs1052536	17	*LIG3*	C	T	0.98
rs1396515*	17	*KCNJ2*	G	C	0.98
rs17608766	17	*GOSR2*	C	T	0.72
rs9892651	17	*PRKCA*	T	C	0.74
rs1805128*	21	*KCNE1*	T	C	7.42

SNP: Single nucleotide polymorphism
*SNPs included in Ion Channel Score
†SNPs excluded by MR-PRESSO method
Weighting for score is per coded allele and reported unaltered from the GWAS [3,4]

Table F. Power calculations for genetic ECG scores

ECG score	Number of SNPs	SD of ECG parameter*	% Variance explained† – R^2	*F*-statistic per SNP‡ – median (range)	Calculated power
P-wave duration	8	12 ms	1.8	43 (35 – 184)	79.6%
PR interval	52	26 ms	5.0	40 (20 – 1110)	99.6%
QT interval	54	23 ms	5.0	47 (24 – 1012)	99.6%

*Standard deviation (SD) estimates from genome wide association studies
†Refers to % variance in measured ECG parameter explained by genetic variants in the score
‡Refers to *F*-statistic per single nucleotide polymorphism (SNP) included in each score. Calculated using ($Beta^2/SE^2$) [9]
Power calculated using online tool (https://sb452.shinyapps.io/power/) for a binary outcome, with sample size 278,792; ratio of cases:controls of 1:15; causal detection effect of 0.85 per SD unit and significance of $P = 0.05$

Table G. SNPs included in atrial fibrillation score

SNP (rsID)	Chromosome	Nearest gene locus	Coded allele	Non-coded allele	Weighting for score (log odds)
rs284277	1	*CASZ1*	C	T	0.0357
rs1545300	1	*KCND3*	G	C	0.0822
rs4073778	1	*CASQ2*	G	A	0.0551
rs79187193	1	*intergenic*	A	G	0.0425
rs6689306	1	*IL6R*	T	C	0.0679
rs11264280	1	*intergenic*	A	G	0.0800
rs72700114	1	*intergenic*	T	C	0.0533
rs72700118	1	*intergenic*	T	G	0.0529
rs577676	1	*intergenic*	C	A	0.0649
rs10753933	1	*PPFIA4*	A	G	0.0507
rs4951258	1	*NUCKS1*	A	C	0.0768
rs7529220	1	*intergenic*	C	T	0.0295
rs2885697	1	*SCMH1*	C	T	0.0322
rs11590635	1	*AGBL4*	C	T	0.0677
rs146518726	1	*intergenic*	T	C	0.1258
rs28387148	2	*GYPC*	G	A	0.0526
rs67969609	2	*ncRNA_TEX41*	A	G	0.1731
rs56181519	2	*intergenic*	G	A	0.1498
rs2288327	2	*TTN*	C	T	0.0327
rs3820888	2	*SPATS2L*	T	C	0.0410
rs35544454	2	*ERBB4*	A	C	0.0924
rs7578393	2	*KIF3C*	T	G	0.1064
rs11125871	2	*USP34*	G	T	0.1784
rs2540949	2	*CEP68*	A	G	0.0784
rs6747542	2	*GMCL1*	C	A	0.0376
rs72926475	2	*intergenic*	C	T	0.0309
rs10804493	3	*PHLDB2*	T	C	0.0369
rs7650482	3	*CAND2*	T	C	0.0995
rs1278493	3	*PPP2R3A*	G	A	0.0327
rs7612445	3	*GNB4*	C	T	0.0553
rs60902112	3	*XXYLT1*	G	T	0.0931
rs73041705	3	*THRB*	G	A	0.0696
rs7373065	3	*intergenic*	G	A	0.0637
rs6790396	3	*SCN10A*	T	C	0.0478
rs34080181	3	*LRIG1*	C	T	0.1196
rs17005647	3	*FRMD4B*	A	G	0.1439
rs6771054	3	*EPHA3*	T	C	0.0406
rs10006327	4	*SLC9B1*	A	G	0.1709
rs244017	4	*intergenic*	C	A	0.3406
rs67249485	4	*intergenic*	G	A	0.0357
rs79399769	4	*intergenic*	C	T	0.0500

SNP (rsID)	Chromosome	Nearest gene locus	Coded allele	Non-coded allele	Weighting for score (log odds)
rs1532170	4	*intergenic*	T	C	0.0669
rs138311480	4	*intergenic*	T	C	0.0342
rs114904067	4	*intergenic*	C	T	0.0439
rs7687819	4	*ALPK1*	C	T	0.0489
rs6829664	4	*CAMK2D*	G	A	0.0500
rs10213171	4	*ARHGAP10*	A	G	0.0441
rs10520260	4	*HAND2-AS1*	T	C	0.1025
rs12648245	4	*intergenic*	G	A	0.0825
rs1458038	4	*intergenic*	A	T	0.1577
rs6596717	5	*intergenic*	T	G	0.0189
rs337705	5	*KCNN2*	A	T	0.0625
rs2012809	5	*intergenic*	A	G	0.0704
rs2040862	5	*WNT8A*	A	G	0.0420
rs6580277	5	*NR3C1*	T	G	0.1010
rs12188351	5	*SLIT3*	T	C	0.0872
rs6891790	5	*intergenic*	C	A	0.0432
rs28439930	5	*intergenic*	T	G	0.0745
rs3951016	6	*SLC35F1*	G	C	0.0409
rs9401451	6	*intergenic*	A	C	0.0476
rs13195459	6	*intergenic*	G	T	0.0403
rs117984853	6	*UST*	G	A	0.0680
rs73366713	6	*ATXN1*	G	T	0.0583
rs34969716	6	*KDM1B*	G	A	0.0371
rs3176326	6	*CDKN1A*	A	G	0.0651
rs2031522	6	*intergenic*	C	T	0.0695
rs11773845	7	*CAV1*	C	T	0.0546
rs55985730	7	*OPN1SW*	A	T	0.1281
rs55734480	7	*DGKB*	A	T	0.0633
rs7789146	7	*KCNH2*	C	A	0.0510
rs6462079	7	*CREB5*	C	T	0.0599
rs35005436	7	*GTF2I*	A	T	0.0723
rs56201652	7	*CDK6*	A	C	0.0493
rs35620480	8	*intergenic*	C	T	0.0246
rs62521286	8	*FBXO32*	T	C	0.0418
rs4871397	8	*intergenic*	G	C	0.0829
rs6994744	8	*PTK2*	T	A	0.0362
rs7508	8	*UTR3/ASAH1*	A	G	0.0344
rs7834729	8	*XPO7*	C	G	0.0397
rs2274115	9	*LHX3*	T	C	0.0446
rs10821415	9	*C9orf3*	A	G	0.0419
rs55693294	10	*NEURL1*	G	T	0.0782
rs11598047	10	*NEURL1*	C	T	0.0576
rs35176054	10	*SH3PXD2A*	G	A	0.0563

SNP (rsID)	Chromosome	Nearest gene locus	Coded allele	Non-coded allele	Weighting for score (log odds)
rs10749053	10	*RBM20*	C	T	0.0977
rs12245149	10	*REEP3*	A	C	0.0270
rs7096385	10	*SIRT1*	T	C	0.0388
rs60212594	10	*SYNPO2L*	G	A	0.1131
rs10458660	10	*C10orf11*	A	G	0.0519
rs4935786	11	*intergenic*	C	T	0.0510
rs76097649	11	*KCNJ5*	G	A	0.0759
rs10741807	11	*NAV2*	C	A	0.0356
rs883079	12	*UTR3/TBX5*	T	C	0.0788
rs10773657	12	*HIP1R*	A	G	0.1536
rs6560886	12	*FBRSL1*	A	G	0.0440
rs4963776	12	*intergenic*	T	C	0.1531
rs17380837	12	*SSPN*	T	A	0.3422
rs12809354	12	*PKP2*	T	C	0.0602
rs11614818	12	*intergenic*	G	C	0.0604
rs2860482	12	*NACA*	G	C	0.0764
rs71454237	12	*intergenic*	G	A	0.0638
rs775498	12	*BEST3*	G	T	0.0716
rs12426679	12	*intergenic*	C	A	0.0330
rs35569628	13	*CUL4A*	T	C	0.0680
rs9506925	13	*intergenic*	G	A	0.0613
rs422068	14	*MYH6*	A	G	0.0389
rs1957021	14	*AKAP6*	G	A	0.0744
rs11156751	14	*AKAP6*	C	G	0.1698
rs73241997	14	*intergenic*	A	C	0.1162
rs2738413	14	*SYNE2*	G	C	0.0658
rs74884082	14	*DPF3*	G	A	0.0548
rs10873298	14	*intergenic*	T	C	0.0465
rs147301839	15	*GCOM1*	G	A	0.0923
rs7170477	15	*HERC1*	T	C	0.1813
rs74022964	15	*intergenic*	T	C	0.1079
rs12908004	15	*intergenic*	C	T	0.0461
rs2759301	15	*ABHD17C*	A	G	0.0711
rs4965430	15	*IGF1R*	C	T	0.0686
rs118159104	16	*CRAMP1*	T	C	0.0615
rs140185678	16	*RPL3L*	A	G	0.0988
rs77316573	16	*PGP*	T	G	0.0428
rs2359171	16	*ZFHX3*	G	A	0.0668
rs72811294	17	*MYOCD*	A	G	0.0200
rs7225165	17	*intergenic*	T	C	0.0383
rs11658278	17	*ZPBP2*	G	A	0.0362
rs1563304	17	*WNT3*	G	A	0.0568
rs9899183	17	*TNFSF12*	G	T	0.0587

SNP (rsID)	Chromosome	Nearest gene locus	Coded allele	Non-coded allele	Weighting for score (log odds)
rs12604076	17	*CYTH1*	G	A	0.1136
rs9953366	18	*SMAD7*	A	C	0.0388
rs8088085	18	*MEX3C*	T	C	0.0872
rs2834618	21	*ncRNA_LINC01426*	G	A	0.0703
rs464901	22	*TUBA8*	T	C	0.0365
rs133902	22	*MYO18B*	T	C	0.0432

SNP: Single nucleotide polymorphism
AF: atrial fibrillation
Weighting for score is per coded allele and reported unaltered from the GWAS as natural log odds [11]

Table H. Baseline characteristics of participants with supraventricular tachycardias in UK Biobank

Characteristic	No supraventricular tachycardias	Supraventricular tachycardias
Number of participants	275908	2884
Age at recruitment (yr)	56.9 ± 8.0	59.6 ± 7.3
Male sex	129751 (47.0)	1507 (52.3)
Body mass index (kg/m^2)	27.4 ± 4.8	27.8 ± 5.0
Coronary Heart Disease	25673 (9.3)	956 (33.2)
Heart Failure	6211 (2.3)	455 (15.8)
Hypertension	91964 (33.2)	1566 (54.3)
Diabetes	18940 (6.9)	386 (13.4)
Atrial fibrillation	17412 (6.3)	1720 (59.6)
‘Lone’ atrial fibrillation	4203 (1.5)	602 (20.9)

Population restricted to unrelated white British ancestry (see **Methods** for full details).
Continuous variables are presented as mean ± standard deviation and categorical variables as number (%).
‘Lone’ atrial fibrillation defined as atrial fibrillation without known coronary heart disease, heart failure, hypertension or diabetes (see **Methods** for full definition).

Table I. Effect of atrial fibrillation genetic risk score on 12-lead ECG parameters

ECG parameter	Change in ECG parameter (ms) (95% CI) *per unit log odds higher AF risk*	*P*-value
**UK Biobank (13,314 participants)**		
P-wave duration	-1.1 (-1.7 to -0.5)	4×10^{-4}
PR interval	-0.4 (-1.4 to 0.5)	0.40
QT_c (Bazett)	-1.1 (-2.0 to -0.2)	0.02

Sensitivity analyses for bidirectional Mendelian randomisation analyses – i.e. effect of genetic risk score for atrial fibrillation (AF) on 12-lead ECG parameters in 13,314 participants with ECG data and no history of AF in UK Biobank. Effect on 12-lead ECG parameter in milliseconds (ms) and 95% confidence intervals (CI) presented per unit log odds (~2.7 OR) increase. *P* calculated using linear regression adjusting for sex, genotyping array and first forty principal components of ancestry.

Table J. Sensitivity analyses for genetically predicted effects of ECG parameters on atrial fibrillation in UK Biobank

	**P-wave duration per 5 ms**		**PR interval per 5 ms**		**QT interval per 5 ms**	
	**OR* (95% CI)**	***P***	**OR* (95% CI)**	***P***	**OR* (95% CI)**	***P***
**UK Biobank – 19,132 cases of AF**						
Primary genetic score	0.91 (0.87-0.96)	2×10^{-4}	0.94 (0.93-0.96)	2×10^{-19}	0.98 (0.97-1.00)	0.02
IVW estimate (fixed-effects)	0.92 (0.88-0.96)	3×10^{-4}	0.94 (0.93-0.96)	3×10^{-19}	0.98 (0.97-0.99)	0.02
IVW estimate (random-effects)	0.92 (0.75-1.12)	0.40	0.94 (0.91-0.98)	1×10^{-3}	0.98 (0.95-1.01)	0.32
Weighted Median MR estimate	0.87 (0.81-0.94)	4×10^{-4}	0.93 (0.91-0.95)	4×10^{-9}	0.99 (0.97-1.01)	0.38
Weighted Mode MR estimate	0.86 (0.79-0.93)	0.006	0.93 (0.91-0.96)	4×10^{-7}	0.98 (0.96-1.00)	0.11
MR-Egger estimate	0.64 (0.45-0.89)	0.009	0.93 (0.86-1.00)	0.06	0.98 (0.92-1.05)	0.57
Egger-intercept test	-	*0.02*	-	*0.69*	-	*0.99*
MR-PRESSO corrected estimate	0.98 (0.86-1.12)	0.79	0.95 (0.93-0.98)	8×10^{-4}	0.99 (0.96-1.02)	0.38

Sensitivity analyses for primary ECG score effects on atrial fibrillation (AF) in UK Biobank. Odds ratios (OR) and 95% confidence intervals (CI) presented per 5ms higher genetic ECG score but (*) not applicable to Egger-intercept test. IVW: Inverse variance weighted; MR: Mendelian Randomisation; MR-PRESSO: Mendelian Randomisation Pleiotropy Residual Sum and Outlier

Table K. Phenoscanner derived details for non-ECG parameter associated traits for SNPs in ECG scores

SNP in score	Reported SNP	r^2 with sentinel	Trait	First author	PMID	P-value
**P-wave duration score**						
rs11689011	rs11894252	0.979	Renal cell carcinoma	Purdue MP	21131975	9.11E-09
rs13242816	rs7801950	0.911	Maternal transmission distortion	Meyer	22377632	6.21E-19
**PR interval score**						
rs12127701	rs12127701	1	LDL	GLGC	24097068	4.54E-36
	rs12127701	1	Total cholesterol	GLGC	24097068	3.87E-26
rs11264339	rs11264339	1	Serum magnesium	Meyer TE	20700443	2.50E-17
rs7638853	rs7638853	1	Expression of SENP2 in brain cortex	Heinzen	19222302	1.83E-09
rs12678719	rs12678719	1	Serum VEGF	Debette S	21757650	1.95E-10
rs4901308	rs8008270	0.964	Prostate cancer	Eeles RA	23535732	2.00E-14
**QT interval score**						
rs12061601	rs12061601	1	Normalized agkistrodon contortrix venom ratio in venous thrombosis	Oudot Mellakh T	22443383	4.53E-16
rs1361754	rs1361754	1	Chromosome Methylation levels in Neural Tissues	Gibbs J	20485568	2.44E-30
	rs1361754	1	Differential expression of PM20D1 probe 2452744 in brain cortex	Heinzen	19222302	2.92E-10
rs1801725	rs1801725	1	Calcium levels	OSeaghdha CM	24068962	9.00E-86
rs7765828	rs6459467	0.996	Blood metabolite levels	Shin SY	24816252	2.00E-16
rs3807375	rs3807375	1	Height	GIANT	25282103	7.20E-11
rs2485376	rs2485376	1	Years of educational attainment	SSGAC	27225129	4.63E-10
rs7122937	rs7122937	1	Chromosome Methylation levels in Neural Tissues	Gibbs J	20485568	2.57E-24
rs174583	rs174583	1	Serum LAPUFA	Kettunen J	22286219	1.33E-267
	rs174583	1	Plasma docosapentaenoic acid levels	Lemaitre RN	21829377	4.17E-147
	rs174583	1	PC aa C363, PC aa C364	Illig T	20037589	6.40E-91
	rs174583	1	Serum ratio of 1- PE (20:4/0:0)/ CHEBI:85670	Suhre K	21886157	3.10E-68
	rs174583	1	LDL	GLGC	24097068	7.00E-41

SNP in score	Reported SNP	r^2 with sentinel	Trait	First author	PMID	P-value
	rs174583	1	Total cholesterol	GLGC	24097068	6.05E-38
	rs174583	1	Triglycerides	GLGC	24097068	8.38E-35
	rs174583	1	HDL	GLGC	24097068	1.19E-26
	rs174583	1	Plasma stearic acid	Wu JH	23362303	9.91E-19
	rs174583	1	Fasting glucose	MAGIC	20081858	1.17E-08
	rs174576	0.979	Differential expression of FADS2 in peripheral blood mononuclear cells	Heinzen	19222302	6.51E-15
	rs174577	0.966	Iron status biomarkers transferrin levels	Benyamin B	25352340	2.00E-17
rs246185	rs246185	1	Height	GIANT	25282103	2.00E-18
	rs246185	1	Age at menarche	ReproGen	25231870	6.80E-16
rs17608766	rs17608766	1	Gene expression of GOSR2 in liver	ICBP	21909115	3.00E-24
	rs17608766	1	Blood pressure	Wain LV	21909110	6.00E-15
	rs17608766	1	Coronary artery disease	Howson JMM	28530674	4.14E-08

Shown are the association results for all single nucleotide polymorphisms (SNPs) in the ECG genetic scores with $r^2>0.8$ (1000 Genomes Phase 3 data) to one of the associated SNPs that achieved genome-wide significance of $P<5\times10^{-8}$ for any biomedical trait (excluding other ECG parameters – P-duration, PR interval, QRS duration, QT duration) as reported in catalogues of associations for European ancestry. Data retrieved via phenoscanner (http://www.phenoscanner.medschl.cam.ac.uk/phenoscanner) [10].

SUPPORTING REFERENCES

1. Christophersen IE, Magnani JW, Yin X, Barnard J, Weng LC, Arking DE, et al. Fifteen genetic loci associated with the electrocardiographic P wave. Circ Cardiovasc Genet. 2017;10(4):e001667. doi: 10.1161/CIRCGENETICS.116.001667. PMID: 28794112.
2. van Setten J, Brody JA, Jamshidi Y, Swenson BR, Butler AM, Campbell H, et al. PR interval genome-wide association meta-analysis identifies 50 loci associated with atrial and atrioventricular electrical activity. Nat Commun. 2018;9(1):2904. doi: 10.1038/s41467-018-04766-9. PMID: 30046033.
3. Arking DE, Pulit SL, Crotti L, van der Harst P, Munroe PB, Koopmann TT, et al. Genetic association study of QT interval highlights role for calcium signaling pathways in myocardial repolarization. Nat Genet. 2014;46(8):826-36. doi: 10.1038/ng.3014. PMID: 24952745.
4. Bihlmeyer NA, Brody JA, Smith AV, Warren HR, Lin H, Isaacs A, et al. Exomechip-wide analysis of 95 626 individuals identifies 10 novel loci associated with QT and jt intervals. Circ Genom Precis Med. 2018;11(1):e001758. doi: 10.1161/CIRCGEN.117.001758. PMID: 29874175.
5. Dayem Ullah AZ, Oscanoa J, Wang J, Nagano A, Lemoine NR, Chelala C. SNPnexus: Assessing the functional relevance of genetic variation to facilitate the promise of precision medicine. Nucleic Acids Res. 2018;46(W1):W109-W13. doi: 10.1093/nar/gky399. PMID: 29757393.
6. Ashburner M, Ball CA, Blake JA, Botstein D, Butler H, Cherry JM, et al. Gene ontology: Tool for the unification of biology. The Gene Ontology Consortium. Nat Genet. 2000;25(1):25-9. doi: 10.1038/75556. PMID: 10802651.
7. The Gene Ontology Consortium. The Gene Ontology resource: 20 years and still going strong. Nucleic Acids Res. 2019;47(D1):D330-D8. doi: 10.1093/nar/gky1055. PMID: 30395331.
8. Mack S, Coassin S, Vaucher J, Kronenberg F, Lamina C, Apo AIVGC. Evaluating the causal relation of APOA-IV with disease-related traits - a bidirectional two-sample Mendelian randomization study. Sci Rep. 2017;7(1):8734. doi: 10.1038/s41598-017-07213-9. PMID: 28821713.
9. Li B, Martin EB. An approximation to the F distribution using the chi-square distribution. Computational Statistics & Data Analysis. 2002;40(1):21-6. doi: 10.1016/s0167-9473(01)00097-4.
10. Staley JR, Blackshaw J, Kamat MA, Ellis S, Surendran P, Sun BB, et al. Phenoscanner: A database of human genotype-phenotype associations. Bioinformatics. 2016;32(20):3207-9. doi: 10.1093/bioinformatics/btw373. PMID: 27318201.
11. Nielsen JB, Thorolfsdottir RB, Fritsche LG, Zhou W, Skov MW, Graham SE, et al. Biobank-driven genomic discovery yields new insight into atrial fibrillation biology. Nat Genet. 2018;50(9):1234-9. doi: 10.1038/s41588-018-0171-3. PMID: 30061737.
12. Verbanck M, Chen CY, Neale B, Do R. Detection of widespread horizontal pleiotropy in causal relationships inferred from Mendelian randomization between complex traits and diseases. Nat Genet. 2018;50(5):693-8. doi: 10.1038/s41588-018-0099-7. PMID: 29686387.
13. Eastwood SV, Mathur R, Atkinson M, Brophy S, Sudlow C, Flaig R, et al. Algorithms for the capture and adjudication of prevalent and incident diabetes in UK Biobank. PLoS ONE. 2016;11(9):e0162388. doi: 10.1371/journal.pone.0162388. PMID: 27631769.
